# Inflammasome Sensor NLRP1 Confers Acquired Drug Resistance to Temozolomide in Human Melanoma

**DOI:** 10.3390/cancers12092518

**Published:** 2020-09-04

**Authors:** Zili Zhai, Jenny Mae Samson, Takeshi Yamauchi, Prasanna K. Vaddi, Yuko Matsumoto, Charles A. Dinarello, Dinoop Ravindran Menon, Mayumi Fujita

**Affiliations:** 1 Department of Dermatology, University of Colorado Anschutz Medical Campus, Aurora, CO 80045, USA; zili.zhai@cuanschutz.edu (Z.Z.); jenny.samson@cuanschutz.edu (J.M.S.); takeshi.yamauchi@cuanschutz.edu (T.Y.); prasanna.vaddi@cuanschutz.edu (P.K.V.); yukomatsumo2525@gmail.com (Y.M.); dinoop.ravindranmenon@cuanschutz.edu (D.R.M.); 2 Department of Medicine, University of Colorado Anschutz Medical Campus, Aurora, CO 80045, USA; cdinare333@aol.com; 3 Department of Veterans Affairs Medical Center, VA Eastern Colorado Health Care System, Aurora, CO 80045, USA; 4 Department of Immunology & Microbiology, University of Colorado Anschutz Medical Campus, Aurora, CO 80045, USA

**Keywords:** melanoma, inflammasome, NLRP1, acquired resistance, chemotherapy

## Abstract

**Simple Summary:**

Acquired drug resistance remains a challenge in the management of cancer patients. Strategies to overcome drug resistance and enhance the response include the combination therapy with agents that target known resistance mechanisms. Inflammasomes are mediators of inflammation. We previously reported the involvement of NACHT, LRR and PYD domains-containing protein (NLRP) 1/3 inflammasomes in the melanoma microenvironment and tumor growth. The aim of this study was to further determine the role of NLRP1 in acquired drug resistance in melanoma. We, for the first time, demonstrate that NLRP1 inflammasome is involved in the development of acquired drug resistance of melanoma. Because drug-tolerant cancer cells become cross-tolerant to other classes of cancer drugs, shared mechanisms could be involved in acquired drug resistance to other drugs.

**Abstract:**

Cancer cells gain drug resistance through a complex mechanism, in which nuclear factor-κB (NF-κB) and interleukin-1β (IL-1β) are critical contributors. Because NACHT, LRR and PYD domains-containing protein (NLRP) inflammasomes mediate IL-1β maturation and NF-κB activation, we investigated the role of inflammasome sensor NLRP1 in acquired drug resistance to temozolomide (TMZ) in melanoma. The sensitivity of melanoma cells to TMZ was negatively correlated with the expression levels of *O*^6^-methylguanine-DNA methyltransferase (MGMT), the enzyme to repair TMZ-induced DNA lesions. When MGMT-low human melanoma cells (1205Lu and HS294T) were treated with TMZ for over two months, MGMT was upregulated, and cells became resistant. However, the resistance mechanism was independent of MGMT, and the cells that acquired TMZ resistance showed increased NLRP1 expression, NLRP inflammasome activation, IL-1β secretion, and NF-κB activity, which contributed to the acquired resistance to TMZ. Finally, blocking IL-1 receptor (IL-1R) signaling with IL-1R antagonist decreased TMZ-resistant 1205Lu tumor growth in vivo. Although inflammation has been associated with drug resistance in various cancers, our paper is the first to demonstrate the involvement of NLRP in the development of acquired drug resistance. Because drug-tolerant cancer cells become cross-tolerant to other classes of cancer drugs, NLRP1 might be a suitable therapeutic target in drug-resistant melanoma, as well as in other cancers.

## 1. Introduction

Despite recent progress in cancer therapeutics, acquired drug resistance remains a challenge in the management of cancer patients [1]. Cancer cells gain drug resistance through a complex mechanism, involving both intrinsic and extrinsic factors in the tumor microenvironment [2,3]. In particular, an inflammatory microenvironment fosters not only tumor development and progression but also drug resistance, and drug treatment further enhances inflammation [2,3,4]. Among the inflammatory conditions involved in acquired drug resistance, the secretion of interleukin-1β (IL-1β) has been implicated as a critical mediator in human cancers [3,5].

We previously reported that IL-1β is spontaneously released from late-stage melanoma cells due to the constitutive activation of nuclear factor-κB (NF-κB) and NACHT, LRR and PYD domains-containing protein (NLRP) inflammasomes [6]. While NF-κB regulates the production of pro-IL-1β, NLRP inflammasomes are responsible for pro-IL-1β cleavage to its biologically active form [7,8]. Among the 14 NLRP protein family members, NLRP3 is the most studied, while NLRP1 is unique because of its caspase recruitment domain (CARD) domain, which allows NLRP1 to directly bind CARD-containing caspases and inhibit melanoma cell apoptosis [9]. Consequently, NLRP1 inflammasomes have been shown to promote tumorigenesis in late-stage melanoma [9] and non-melanoma skin cancers [10].

NLRP1 inflammasomes activate the NF-κB pathway [9], a known tumor promoter. NF-κB activation renders cancers resistant to platinum-based anticancer drugs, topoisomerase inhibitors, and DNA alkylating agents, including cisplatin, etoposide, and temozolomide (TMZ) [11,12,13]. TMZ is an oral chemotherapy drug used in the clinic as the first-line treatment for glioblastoma multiforme (GBM), the second-line treatment for astrocytoma, and an off-label treatment for melanoma. However, TMZ has not shown remarkable improvement in the overall and progression-free survival of melanoma patients [13]. Melanoma cells are intrinsically resistant to TMZ due to the expression of *O*^6^-methylguanine-DNA methyltransferase (MGMT), the enzyme responsible for repairing DNA lesions induced by TMZ [14,15]. Accordingly, pharmacological depletion or inhibition of MGMT has demonstrated enhanced TMZ sensitivity in pre-clinical studies on GBM and melanoma [16]. Nonetheless, GBM cells with low levels of MGMT still show resistance to TMZ [17,18], suggesting that MGMT-independent mechanisms are also involved in the initial or acquired resistance to TMZ.

We speculated that cancer cells with low levels of MGMT could be used to study MGMT-independent mechanisms because these cells are initially sensitive to TMZ but then acquire MGMT-independent resistance mechanisms after drug exposure. The functions of NLRP1 as both a melanoma tumor promotor and an apoptosis suppressor lead us to hypothesize that NLRP1 plays a role in the development of acquired drug resistance in human melanoma. In this paper, we provide evidence that depicts NLRP1 as a potential therapeutic target in drug-resistant melanoma to TMZ. Although inflammation and activated NF-κB signaling have been associated with drug resistance in various cancers [11,12,13,19], only one paper reported the involvement of NLRP3 in the sensitivity of oral squamous cell carcinoma to 5-fluorouracil [20]. However, the paper failed to study the role of NLRP3 in acquired drug resistance, a critical issue in medical oncology. Our paper is the first to demonstrate the involvement of NLRP in the development of acquired drug resistance. A recent paper demonstrated that, after drug exposure, drug-tolerant cancer cells become cross-tolerant to other classes of cancer drugs [21], suggesting that shared mechanisms could be involved in acquired drug resistance to other drugs.

## 2. Results

### 2.1. Human Melanoma Cell Lines That Express Low Levels of MGMT Are Initially Sensitive to TMZ

**Figure 1:**
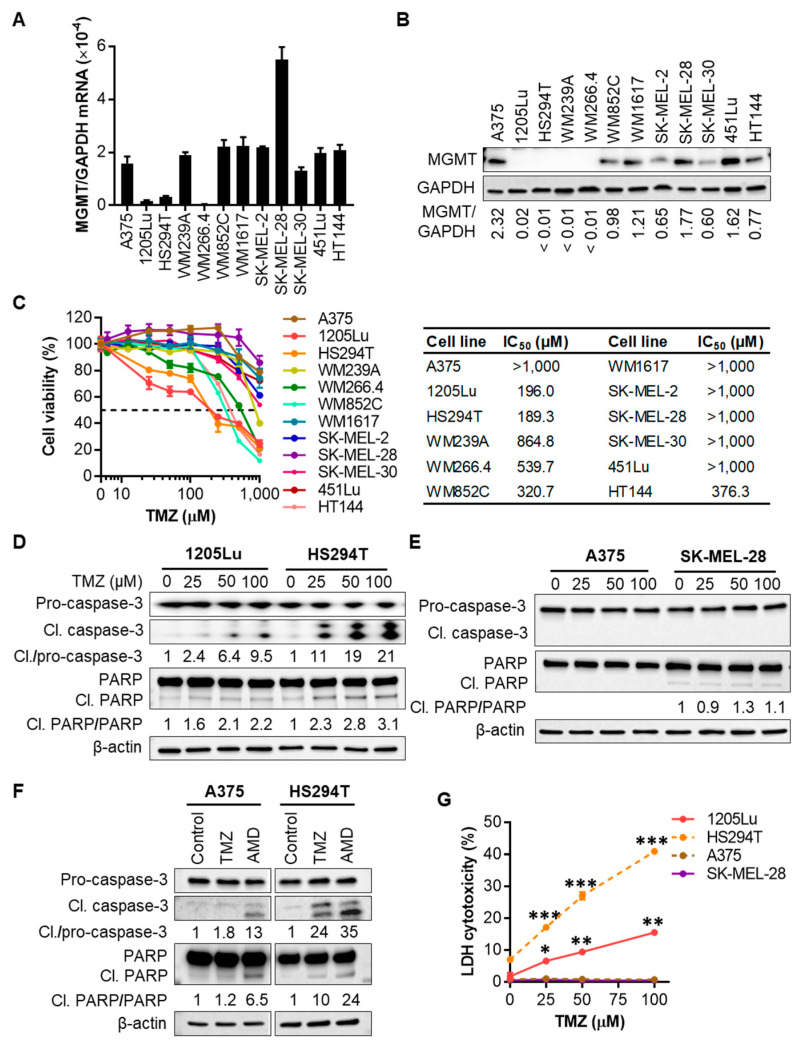
Melanoma cell lines with low expression levels of *O*^6^-methylguanine-DNA methyltransferase (MGMT) are sensitive to temozolomide (TMZ). (**A**) qRT-PCR analysis of MGMT mRNA expression in a panel of 12 human metastatic melanoma cell lines. (**B**) Western blot analysis of MGMT protein expression in 12 metastatic melanoma cell lines. The band densities of MGMT were digitally quantitated and normalized to those of the corresponding glyceraldehyde-3-phosphate dehydrogenase (GAPDH) in each cell line. (**C**) Melanoma cell viability evaluated by 3-(4,5-dimethylthiazol-2-yl)-5-(3-carboxymethoxyphenyl)-2-(4-sulfophenyl)-2H-tetrazolium (MTS) assay after the treatment with a single dose of TMZ at 0-1 mM for 72 h. Fifty percent inhibitory concentration (IC_50_) values were calculated using GraphPad Prism software. (**D**) Western blot analysis of caspase-3 cleavage and Poly(ADP-ribose) polymerase (PARP) cleavage indicated by the presence of cleaved (17, 19 kDa) caspase-3, and cleaved (89 kDa) PARP, respectively, in 1205Lu and HS294T cells treated with TMZ at 0–100 µM for 48 h. The band densities of cleaved caspase-3 and PARP were digitally quantitated and normalized in each cell line to those of the corresponding pro-forms subjected to the same treatment. (**E**) Western blot analysis of caspase-3 and PARP cleavages in A375 and SK-MEL-28 cells. (**F**) Western blot analysis of caspase-3 and PARP cleavages in A375 and HS294T cells treated with 100 µM TMZ for 48 h or apoptosis inducer actinomycin D (AMD; 10 µM) for 6 h. (**G**) Lactate dehydrogenase (LDH) release into culture medium measured using a colorimetric assay as a marker of cell death after TMZ treatment for 48 h. Data are representative of two independent experiments, and are expressed as the mean ± SEM (*n* = 3–4). * *p* < 0.05, ** *p* < 0.01, and *** *p* < 0.001 vs. the corresponding vehicle control.

MGMT contributes to the intrinsic and acquired resistance of tumor cells to TMZ through transcriptional induction and increased enzyme activity [15]. However, GBM cells with low levels of MGMT still show resistance to TMZ [17,18]. Therefore, to understand MGMT-independent acquired resistance mechanisms to TMZ, we selected melanoma cells that were initially sensitive and became resistant later. We first assessed MGMT mRNA and protein levels and used them as a biomarker for intrinsic TMZ resistance in our panel of 12 human metastatic melanoma cell lines. Among them, 1205Lu, HS294T, and WM266.4 cells consistently displayed the lower expression of MGMT mRNA and protein (Figure 1A,B and Figure S1). Cell viability assays of all 12 cell lines confirmed that MGMT-low cell lines (1205Lu, HS294T, and WM266.4) responded initially to TMZ treatment, with 50% inhibitory concentration (IC_50_) values of ~200 (1205Lu and HS294T) or 540 µM (WM266.4) (Figure 1C). Though WM852C and HT144 had IC_50_ values between 300–400 µM, they, like other MGMT-high cell lines, including A375, SK-MEL-28, and WM1617, did not respond to the therapeutic concentrations of TMZ (close to 100 μM maximum [22,23]) (Figure 1C). Therefore, in the following experiments, we tested our hypothesis using the MGMT-low 1205Lu and HS294T cells.

TMZ has been known to induce apoptosis in human melanoma cell lines [23,24], which occurs 72–96 h after TMZ treatment [23,25]. To investigate whether decreased cell viability of MGMT-low 1205Lu and HS294T cells is related to TMZ-mediated apoptotic event, we evaluated caspase-3 cleavage 48 h after TMZ treatment. Western blot analysis of cell lysates demonstrated that TMZ at 25–100 μM induced caspase-3 cleavage in 1205Lu and HS294T cells in a dose-dependent manner (Figure 1D and Figure S2). Poly(ADP-ribose) polymerase (PARP) senses and repairs DNA damage in response to alkylating agents and is a cleavage target of caspase-3 during apoptosis [26,27]. We also found increased levels of the cleaved 89 kDa fragment of PARP in TMZ-treated 1205Lu and HS294T cells (Figure 1D and Figure S2). On the other hand, TMZ treatment did not induce caspase-3 cleavage and PARP activation in MGMT-high A375 and SK-MEL-28 cells (Figure 1E and Figure S3). The differential TMZ-induced apoptosis in MGMT-low cells (1205Lu and HS294T) and MGMT-high cells (A375 and SK-MEL-28) were confirmed using actinomycin D as a positive control for inducing apoptosis. While either TMZ or actinomycin D induced the cleavage of caspase-3 and PARP in HS294T cells, the effects were minimal when A375 cells were treated with TMZ (Figure 1F and Figure S4). Consistent with these data, TMZ-induced cell death was observed in 1205Lu and HS294T but not in MGMT-high A375 and SK-MEL-28 cells (Figure 1G). Altogether, these data suggest that MGMT-low melanoma cells are sensitive to TMZ treatment.

### 2.2. TMZ Enhances NLRP1 and NLRP3 Expression, Activates NLRP Inflammasomes, and Induces IL-1β Secretion in MGMT-Low Human Melanoma Cells

**Figure 2:**
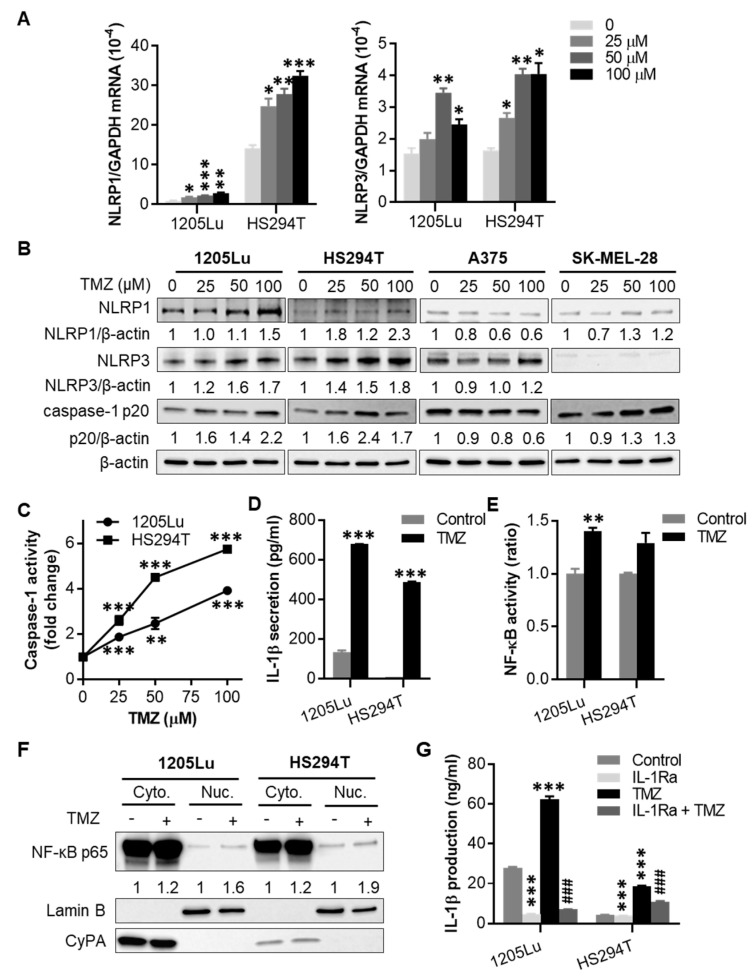
Effects of TMZ on NACHT, LRR and PYD domains-containing protein (NLRP) expression and inflammasome activation. Where indicated, cells were treated with a single dose of TMZ for 48 h, then tested for (**A**) NLRP1 and NLRP3 mRNA expression by qRT-PCR, (**B**) NLRP1 and NLRP3 protein expression, as well as cleaved caspase-1 (p20 form) by Western blot, and (**C**) caspase-1 activity by fluorochrome-labeled inhibitors of caspases (FLICA) apoptosis assay. In (**B**), the band densities were quantitated and normalized to those of the corresponding loading control β-actin subjected to the same treatment. (**D**) Interleukin-1β (IL-1β) secretion analyzed by enzyme-linked immunosorbent assay (ELISA) from cells treated with 100 µM TMZ for 48 h. (**E**) Nuclear factor-κB (NF-κB) activity in cells treated with 100 μM TMZ for 48 h, determined using the Ready-to-Glow Secreted luciferase assay. (**F**) Western blot analysis of intracellular localization of NF-κB p65 in cells treated with 100 μM TMZ for 48 h. Cytoplasmic and nuclear fractions of cells were isolated and assayed for NF-κB p65 expression. Cyclophilin A (CyPA) and Lamin B were used as markers for cytoplasmic and nuclear proteins, respectively. The band densities of NF-κB p65 were quantitated and normalized to those of the corresponding loading control. (**G**) Intracellular IL-1β production analyzed by ELISA from cells treated with 100 µM TMZ and/or 10 µg/mL IL-1 receptor antagonist (IL-1Ra) for 48 h. Data are representative of two independent experiments, and are expressed as the mean ± SEM (*n* = 3). * *p* < 0.05, ** *p* < 0.01, and *** *p* < 0.001 vs the corresponding vehicle control. ^###^ *p* < 0.001 vs the TMZ alone treatment.

We tested the TMZ effects on inflammasomes in TMZ-sensitive 1205Lu and HS294T cells. When they were treated with one dose of TMZ for 48 h, we observed a significant increase in mRNA and protein expression of inflammasome sensors NLRP1 and NLRP3, as well as the cleavage of inflammasome effector caspase-1 in both cell lines (Figure 2A,B and Figure S5). In contrast, TMZ had little to no impact on NLRP1 and NLRP3 expression, as well as caspase-1 cleavage in TMZ-resistant A375 and SK-MEL-28 cells (Figure 2B and Figure S5). Because both NLRP1 and NLRP3 form inflammasomes upon activation, we then tested whether TMZ induces inflammasome activation. Following TMZ treatment for 48 h, caspase-1 activity (Figure 2C) and IL-1β secretion (Figure 2D) were increased in both cell lines. These results demonstrate that TMZ enhances NLRP1 and NLRP3 expression, activates NLRP inflammasomes, and induces IL-1β secretion in MGMT-low human melanoma cells.

Chemotherapy-mediated genotoxic stress has been shown to induce NF-κB activation and inflammatory response [28]. Because IL-1β secretion activates NF-κB via IL-1 receptor (IL-1R) signaling, we studied NF-κB activation. We found that TMZ not only induced IL-1β secretion (Figure 2D) but also enhanced cellular NF-κB activity (Figure 2E), NF-κB p65 nuclear translocation (Figure 2F and Figure S6), and IL-1β production (Figure 2G) in 1205Lu and HS294T cells. As expected, inhibiting IL-1R signaling with IL-1R antagonist (IL-1Ra) reduced the effect of TMZ on IL-1β production in these cells. On the other hand, TMZ-resistant MGMT-high A375 and SK-MEL-28 cells did not show enhanced NF-κB activity in response to TMZ treatment (Figure S7). Further analysis of intracellular IL-1β production showed no changes in A375 cells following TMZ treatment for 48 h (Figure S8). These data suggest that TMZ-sensitive and -resistant melanoma cells exhibit distinct characteristics of inflammation and hence respond differently to TMZ treatment.

### 2.3. TMZ-Mediated Initial Inflammasome Activation Is NLRP1- and NLRP3-Dependent

**Figure 3:**
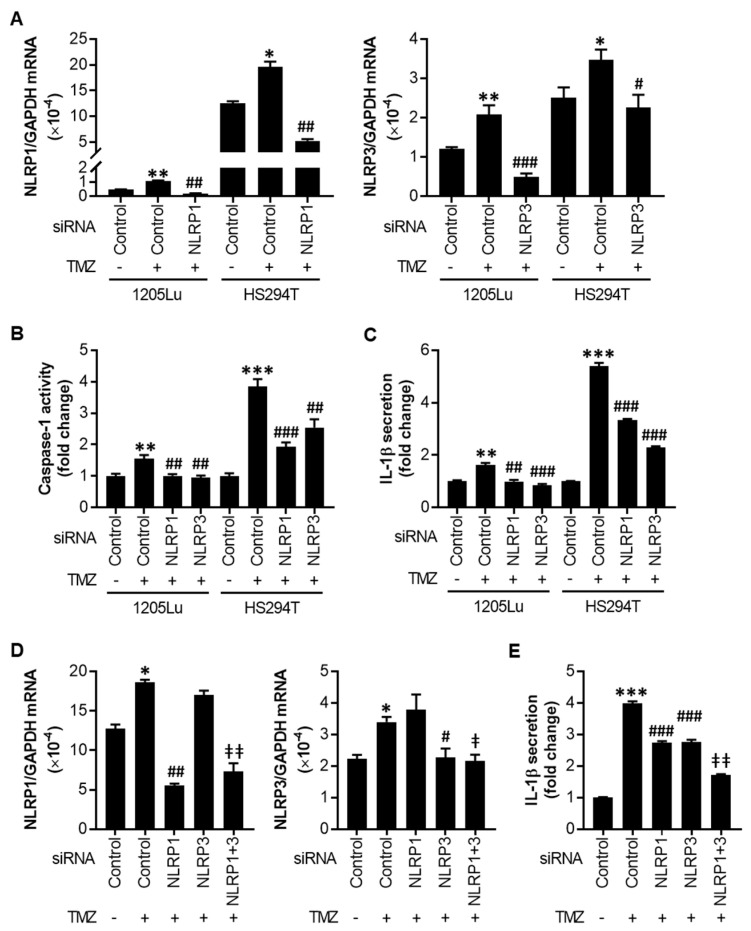
Effects of NLRP1 and NLRP3 small interfering RNAs (siRNAs) on inflammasome activation. Cells were transfected with siRNA control (siControl), siNLRP1, and/or siNLRP3 (40 nM each) for 18 h, then treated with 100 μM TMZ. (**A**) qRT-PCR analysis of NLRP1 (left) and NLRP3 (right) in 1205Lu and HS294T cells at 24 h after treatment with TMZ. (**B**) Caspase-1 activity and (**C**) IL-1β secretion in 1205Lu and HS294T cells at 48 h after treatment with TMZ. (**D**) qRT-PCR analysis of NLRP1 and NLRP3 in HS294T cells at 24 h after TMZ treatment. (**E**) IL-1β secretion in HS294T cells at 48 h after TMZ treatment. Data are expressed as the mean ± SEM (*n* = 3–4). * *p* < 0.05, ** *p* < 0.01, and *** *p* < 0.001 vs. the corresponding vehicle control plus negative control siRNA (**A**–**E**). ^#^ *p* < 0.05, ^##^ *p* < 0.01, and ^###^ *p* < 0.001 vs. the corresponding negative control siRNA plus TMZ treatment (**A**–**E**). ^ǂ^ *p* < 0.05 and ^ǂǂ^ *p* < 0.01 vs the corresponding either siNLRP1 or siNLRP3 (**D**) or both siNLRP1 and siNLRP3 (**E**).

Next, to evaluate the relationship between the increased NLRP1/3 expression and activated inflammasomes observed in Figure 2, we silenced NLRP1 or NLRP3 in melanoma cells using small interfering RNAs (siRNAs) and treated the cells with 100 µM TMZ for 48 h (Figure 3A). While knocking down either NLRP1 or NLRP3 reversed the TMZ-mediated increase in caspase-1 activity and IL-1β secretion to the levels without TMZ in 1205Lu cells, the effects were less in HS294T cells (Figure 3B,C). Therefore, we performed a dual knockdown of NLRP1 and NLRP3 in HS294T cells (Figure 3D) and found an additive effect of NLRP1 and NLRP3 on IL-1β secretion (Figure 3E). These data demonstrate that both NLRP1 and NLRP3 contribute to TMZ-mediated inflammasome activation.

### 2.4. After Chronic Exposure to TMZ, Human Melanoma Cells Display Acquired Resistance to TMZ through Activation of NLRP1 Inflammasomes and IL-1β Secretion

**Figure 4:**
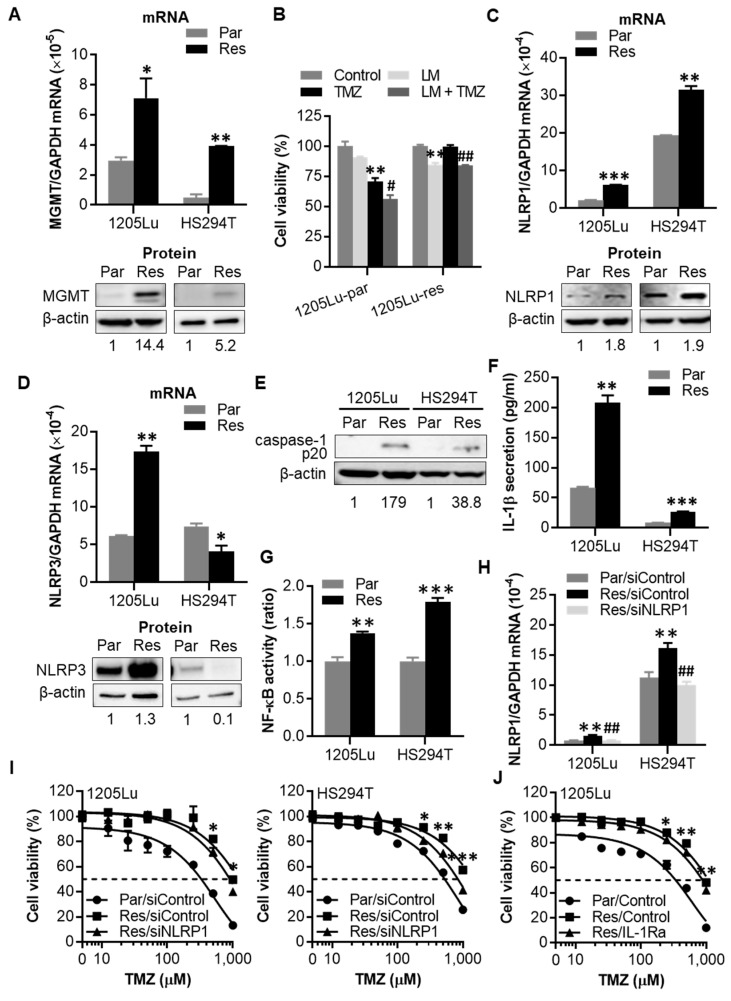
TMZ-resistant cells show increased NLRP1 expression and IL-1β secretion levels. Resistant 1205Lu and HS294T cells were generated after exposing cells to stepwise increasing doses of TMZ (50–200 μM for 1205Lu and 25–100 μΜ for HS294T) over two months. (**A**) qRT-PCR analysis of MGMT mRNA expression (upper panel) and Western blot analysis of MGMT protein expression (lower panel) in resistant cells. (**B**) Cell viability of 1205Lu parental and resistant cells evaluated by MTS assay after the treatment with a single dose of TMZ and/or a single dose of the MGMT inhibitor Lomeguatrib (LM) for 72 h. (**C**) NLRP1 mRNA (upper panel) and protein (lower panel) expression, (**D**) NLRP3 mRNA (upper panel) and protein (lower panel) expression, (**E**) caspase-1 cleavage, (**F**) IL-1β secretion, and (**G**) NF-κB activity of resistant 1205Lu and HS294T cells. (**H**) NLRP1 mRNA expression in parental and resistant 1205Lu and HS294T cells at 24 h after transfection with siNLRP1. (**I**) Cell viability of parental and resistant 1205Lu (left) and HS294T (right) cells after NLRP1 knockdown for 18 h and then TMZ treatment for 72 h. (**J**) Cell viability of resistant 1205Lu cells after daily treatment with IL-1Ra (10 μg/mL) for 72 h. Parental and resistant cells are indicated by “Par” and “Res”, respectively. In (**I**,**J**), dashed lines indicate the 50% inhibitory concentrations (IC_50_) of TMZ. Data are representative of three independent experiments, and are expressed as the mean ± SEM (*n* = 3-4). * *p* < 0.05, ** *p* < 0.01, and *** *p* < 0.001 vs the corresponding parental cells (**A**),(**C**),(**D**),(**F**),(**G**), the corresponding control cells (**B**), the Res/siControl cells (**I**), or the Res/Control cells (**J**). ^#^ *p* < 0.05 and ^##^ *p* < 0.01 vs the TMZ alone cells (**B**) or the Res/siControl cells (**H)**.

To understand the role of increased NLRP1/3 expression and activated NLRP inflammasomes in acquired chemoresistance, TMZ-resistant 1205Lu and HS294T cells were generated by growing cells in TMZ-containing medium for over two months. TMZ resistance was confirmed by increasing IC_50_ and expression of MGMT, a key marker for TMZ resistance (Figure 4A and Figure S9) [14,29]. The resistance phenotype was sustained even following freeze-thaw cycles (Figure S10), demonstrating the stability of acquired TMZ resistance. Interestingly, while the combination of Lomeguatrib, an MGMT inhibitor, and TMZ showed an additive inhibitory effect on cell growth in parental cells, no additive effects were observed in resistant cells (Figure 4B), indicating the involvement of an MGMT-independent mechanism in TMZ resistance. Similar to the response to a single TMZ dose, NLRP1 mRNA and protein expression levels were significantly increased in both 1205Lu and HS294T resistant cells (Figure 4C and Figure S11). However, increased NLRP3 mRNA and protein expression levels were seen only in 1205Lu but not in HS294T resistant cells (Figure 4D and Figure S12), indicating that NLRP1 but not NLRP3 plays a crucial role in acquired TMZ-resistant mechanisms. Together with the increased NLRP1 expression, both resistant cell lines showed an increase in caspase-1 cleavage, IL-1β secretion, and NF-κB activity (Figure 4E–G and Figure S13).

Next, in order to understand whether the increased NLRP1 and inflammasome activation are causative or consequential to acquired TMZ resistance, we evaluated the effects of NLRP1 knockdown on TMZ resistance. We found that whereas knocking down NLRP1 (Figure 4H) did not alter the viability of resistant 1205Lu and HS294T cells (Figure S14) it enhanced their sensitivity to high doses of TMZ (Figure 4I). Because active NLRP1 inflammasomes induce IL-1R signaling activation through IL-1β secretion, we blocked IL-1R signaling using IL-1Ra. Treatment of melanoma cells with IL-1Ra reduced the IC_50_ value of TMZ in resistant 1205Lu cells (Figure 4J), while IL-1Ra had no effect on the viability of these resistant cells in the absence of TMZ (Figure S15). In contrast, IL-1Ra did not affect the sensitivity of primarily resistant SK-MEL-28 cells to TMZ treatment (Figure S16). These findings demonstrate at least a partial involvement of NLRP1 expression and activated NLRP1 inflammasomes in acquired TMZ resistance.

### 2.5. TMZ-Induced NLRP1 Inflammasome Activation Functions Downstream of Notch Signaling

**Figure 5:**
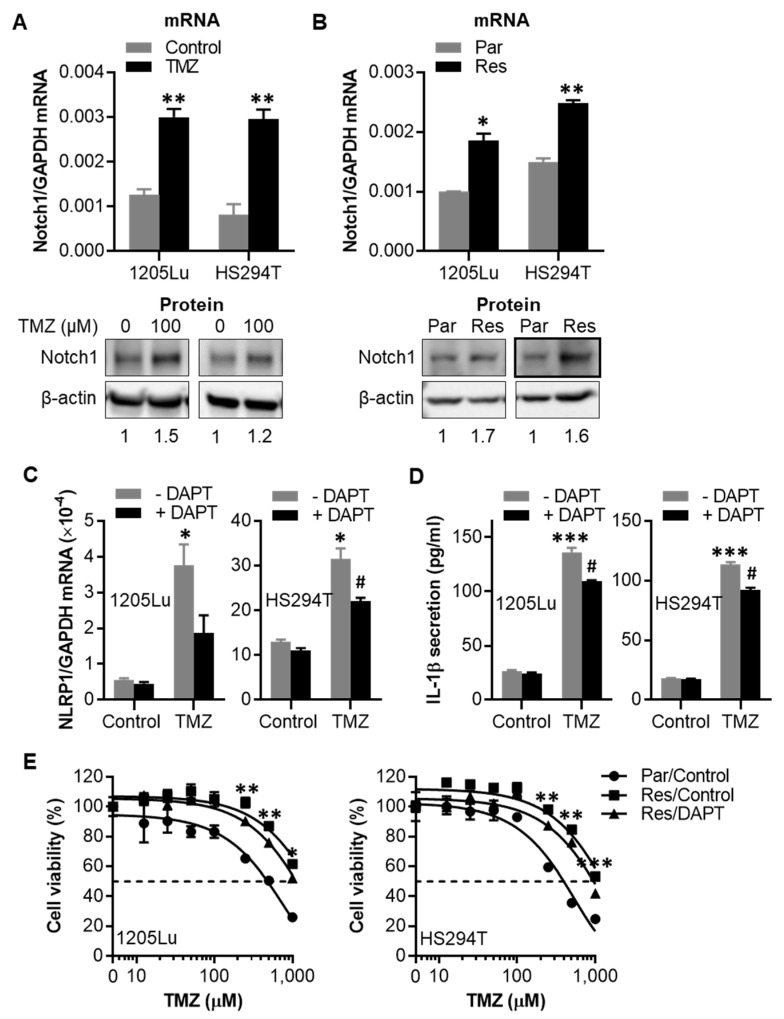
Effects of Notch1 inhibitor *N*-[*N*-(3,5-difluorophenacetyl-L-alanyl)]-(*S*)-phenylglycine *t*-butyl ester (DAPT) on NLRP1 gene expression, IL-1β secretion, and cell susceptibility to TMZ. (**A**) qRT-PCR (upper panel) and Western blot (lower panel) assays of Notch1 in parental 1205Lu and HS294T cells treated with 100 µM TMZ for 48 h. (**B**) qRT-PCR (upper panel) and Western blot (lower panel) assays of Notch1 in parental and resistant 1205Lu and HS294T cells. The band densities of Notch1 in (**A**) and (**B**) were quantitated and normalized to those of the corresponding loading control β-actin. (**C**) qRT-PCR analysis of NLRP1 mRNA expression and (**D**) ELISA analysis of secreted IL-1β in 1205Lu (left panel) and HS294T (right panel) cells treated with 5 μM DAPT and/or 100 μM TMZ for 48 h. (**E**) Cell viability of parental and resistant 1205Lu (left panel) and HS294T (right panel) cells measured by MTS assay after treatment with TMZ ± 10 μM DAPT for 72 h. Dashed lines indicate the 50% inhibitory concentrations (IC_50_) of TMZ. Resistant cells are indicated by “Res”, while parental cells by “Par”. Data are representative of three independent experiments and are expressed as the mean ± SEM (*n* = 3). * *p* < 0.05, ** *p* < 0.01, and *** *p* < 0.001 vs the untreated control (**A**,**C,D**), the corresponding parental cells (**B**), or the Res/Control (**E**). ^#^ *p* < 0.05 vs the TMZ alone treatment.

Notch signaling is involved in crosstalk with other oncogenic signaling pathways, including IL-1β and NF-κB, and plays a role in tumor survival and chemoresistance [30,31]. We found that a single dose of TMZ treatment enhanced the mRNA and protein expression of Notch1 in 1205Lu and HS294T parental cells (Figure 5A and Figure S17). Similarly, Notch1 expression levels were found to be upregulated in TMZ-resistant cells (Figure 5B and Figure S18).

Considering the crosstalk between the Notch and IL-1β signaling pathways, we explored the relationship between Notch, NLRP1, and IL-1β. Inhibiting IL-1β signaling using IL-1Ra or silencing NLRP1 had no effect on TMZ-induced Notch1 mRNA expression in 1205Lu and HS294T parental cells (Figure S19), indicating neither NLRP1 itself nor NLRP1 inflammasomes are required for TMZ-mediated Notch1 upregulation.

However, inhibiting the Notch pathway with *N*-[*N*-(3,5-difluorophenacetyl-L-alanyl)]-(*S*)-phenylglycine *t*-butyl ester (DAPT), a classical Notch signaling inhibitor that suppresses the proteolytic cleavage and activation of Notch by specific inhibition of γ-secretase [30], reduced both TMZ-induced upregulation of NLRP1 gene expression and IL-1β secretion in 1205Lu and HS294T parental cells (Figure 5C,D), and sensitized resistant cells to TMZ treatment (Figure 5E). These data demonstrate that TMZ-induced NLRP1 expression and inflammasome activation are regulated by Notch signaling.

### 2.6. TMZ Enhances the Expression of NLRP1, IL-1β, and Notch1 in 1205Lu Tumors

**Figure 6:**
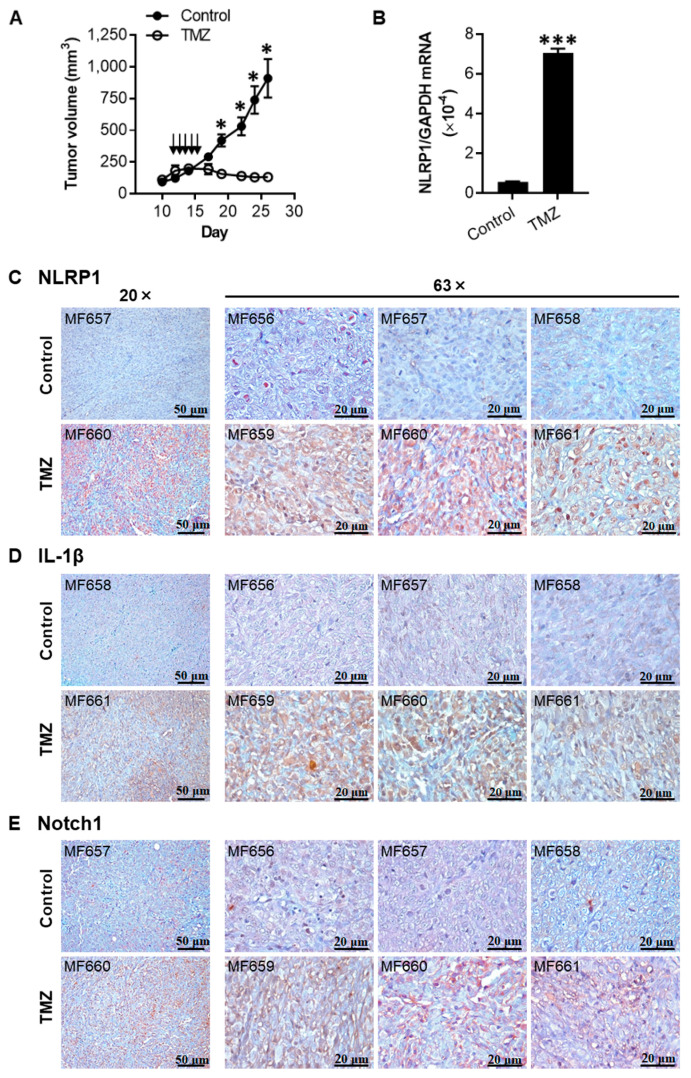
TMZ inhibits tumor growth and enhances the expression of NLRP1, IL-1β, and Notch1 in 1205Lu tumors in vivo. (**A**) Tumor growth curve of 1205Lu parental cells injected subcutaneously in mice, intraperitoneally treated with a TMZ cycle or 10% dimethyl sulfoxide (DMSO) (Control) (indicated by arrows). Tumor growth was monitored for 26 days. Data are expressed as the mean ± SEM. *n* = 10 tumors (control) or 12 tumors (TMZ). (**B**) qRT-PCR analysis of NLRP1 expression in single tumor cells isolated from the tumor tissues studied in (**A**). Data are expressed as the mean ± SEM. *n* = 3 samples. * *p* < 0.05 and *** *p* < 0.001 vs the corresponding tumors. (**C**–**E**) Immunohistochemical staining of tumor tissues with NLRP1 (**C**), IL-1β (**D**), and Notch1 (**E**). Samples MB656, MB657, and MB658 were from the control group, whereas MB659, MB660, and MB661 were from the TMZ group. Bar = 50 μm (left panels, 20×) or 20 μm (right panels, 63×). Representative images are shown.

Next, to evaluate whether the effects of TMZ observed in vitro were reproducible in vivo, we subcutaneously injected 1205Lu parental cells into nude mice and treated the mice with TMZ daily for five days. The dosage and regimen used were based on the clinical usage of TMZ to treat human melanoma patients [22]. In line with the in vitro data showing that 1205Lu parental cells were sensitive to TMZ (Figure 1C), the tumor growth was inhibited in mice treated with TMZ when compared to animals receiving only vehicle (Figure 6A). NLRP1 mRNA expression levels were significantly upregulated in the tumor cells from TMZ-treated animals compared to those from control mice (Figure 6B). Immunohistochemical analyses of tumors confirmed increased protein levels of NLRP1, IL-1β, and Notch1 in tumor cells in mice receiving TMZ treatment compared with those receiving vehicle control (Figure 6C–E), demonstrating that TMZ induced similar biological effects on tumor cell growth, Notch signaling, and NLRP1 inflammasome activation in vivo as observed in 2D cell culture.

### 2.7. IL-1Ra Inhibits Tumor Growth of Melanoma Cells That Have Acquired TMZ Resistance

**Figure 7:**
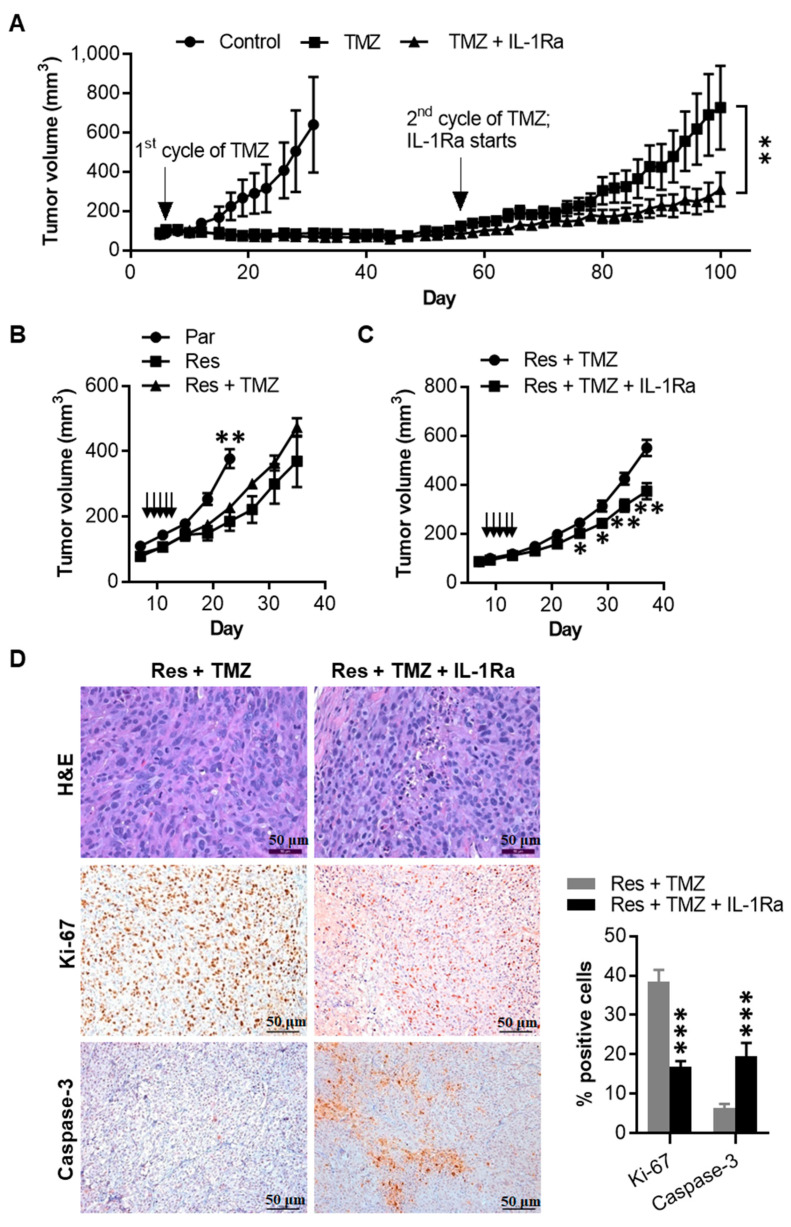
IL-1Ra inhibits TMZ-resistant 1205Lu tumor growth in vivo. (**A**) Tumor growth curve of 1205Lu parental cells subcutaneously injected in mice, treated with a TMZ cycle or 10% DMSO (Control) intraperitoneally (indicated by arrow), and further treated with a second cycle of TMZ together with saline or IL-1Ra (daily subcutaneous injection, starting with the second cycle of TMZ and being kept until the end of the experiment on day 100). Data are expressed as the mean ± SEM. n = 4 tumors (Control), 10 tumor (TMZ), or 10 tumors (TMZ + IL-1Ra). (**B**) Tumor growth curve of 1205Lu parental cells (Par), resistant cells (Res) injected subcutaneously in mice, treated with a cycle of TMZ or 10% DMSO as vehicle control (daily intraperitoneally for five days, indicated by arrows). Tumor growth was monitored for 23 or 35 days (parental or resistant tumors, respectively). Data are expressed as the mean ± SEM. *n* = 8 tumors per each group. (**C**) Tumor growth curve of 1205Lu resistant cells (Res) injected subcutaneously in mice, treated with a cycle of TMZ (daily intraperitoneally for five days, indicated by arrows) together with saline or IL-1Ra (daily subcutaneously, starting with TMZ and being kept until the end of the experiment on day 37). Data are expressed as the mean ± SEM. *n* = 8 tumors (Res + TMZ) or 14 tumors (Res + TMZ + IL-1Ra). (**D**) Staining of tumor sections from (**C**) tumors with hematoxylin and eosin (H&E) (upper panel), Ki-67 (middle panel), and caspase-3 (lower panel). Ki-67-positive cells and caspase-3-positive cells were counted in the whole field under a microscope and presented as % positive tumor cells. Bar = 50 μm (H&E) or 100 μm (Ki-67 and caspase-3). Representative images are shown or data are expressed as the mean ± SEM (*n* = 5). * *p* < 0.05, ** *p* < 0.01, and *** *p* < 0.001 vs the corresponding or indicated tumors (**A**,**C**,**D**) or the combined resistant tumors at the same time point (**B**).

We evaluated whether TMZ induces acquired resistance in vivo (Figure 7A). Similar to the experiments shown in Figure 6A, we injected 1205Lu parental cells subcutaneously into nude mice and treated the mice with one cycle of TMZ (15 mg/kg × five days). After observing significant growth inhibition, we kept monitoring tumor growth in mice without sacrificing them. Around 50 days after the initial cycle of TMZ treatment, tumors started to regrow in mice. When the tumor volume reached 100 mm^3^, the mice were randomly assigned into two treatment groups: TMZ alone (2nd cycle of TMZ for five days) or combination (2nd cycle of TMZ for five days plus daily treatment of IL-1Ra). Interestingly, the regrown tumors kept growing despite the second cycle of TMZ treatment, whereas their growth was inhibited when mice received the combination of TMZ and IL-1Ra, further demonstrating the requirement of IL-1R signaling for acquiring drug resistance.

Finally, we evaluated whether the TMZ-resistant phenotype observed in vitro were reproducible in vivo. Although resistant tumors grew slower than parental tumor cells, the 5-day TMZ treatment did not affect the tumor growth of 1205Lu resistant cells (Figure 7B), suggesting the resistant phenotype remained stable in vivo. Next, we used IL-1Ra in conjunction with the 5-day TMZ regimen. This combination treatment reduced the tumor growth of 1205Lu resistant cells (Figure 7C), confirming that IL-1R signaling is involved in acquired resistance to TMZ in melanoma. In line with the tumor growth change, hematoxylin and eosin staining revealed decreased mitosis and increased pyknotic cells in tumors treated with the combination of IL-1Ra and TMZ compared to those treated with TMZ only (Figure 7D). Similarly, immunohistochemical analyses demonstrated decreased tumor cell proliferation (Ki-67-positive cells) but increased apoptosis (caspase-3-positive cells) in tumors from mice receiving the combination treatment compared with those receiving TMZ alone (Figure 7D). These data indicate that the TMZ-resistant phenotype we observed in vitro is reproducible in vivo, and acquired resistant tumor growth is IL-1R signaling dependent.

## 3. Discussion

The mechanisms of drug resistance in melanoma are diverse and complex. They include the acquisition of stem-like phenotypes [32], induction of tumor secretomes [33], activation of alternative signaling pathways [33], and the occurrence of epithelial–mesenchymal-like transitions [32]. Our finding that NLRP inflammasome is involved in TMZ resistance may suggest a novel role of NLRP proteins or inflammasome complex as a part of the drug-induced network of altered signals.

The sensitivity of melanoma cells to TMZ has been negatively correlated with their MGMT expression levels [23,25]. Consistent with these reports, we found MGMT-high melanoma cells were intrinsically resistant to TMZ. Similarly, MGMT expression levels were increased when MGMT-low melanoma cells became resistant to TMZ, suggesting that increased DNA repair supported the acquired resistance to the DNA alkylating agent in these cells. However, despite upregulated MGMT expression, the resistant mechanism was independent of MGMT, and the cells that acquired TMZ resistance showed increased NLRP1 inflammasome activation, NF-κB activity, and IL-1β secretion, which contributed to the melanoma cells’ resistance to TMZ. This increased inflammatory response in the acquired resistant cells was not only demonstrated in vitro but also confirmed in vivo. Although local inflammation and activated NF-κB signaling have been associated with TMZ treatment and resistance in both GBM and melanoma [13,34,35], the involvement of NLRP or NLRP inflammasomes has not been nowadays reported in the development of drug resistance in any cells. NLRP1 participates not only in inflammasome activation but also in the negative regulation of apoptosis through its C-terminal CARD domain [36,37]. These two distinct properties of NLRP1 have been shown to play integral roles in the promotion of tumor growth and survival in human melanoma [9], and, therefore, it was not surprising to discover that NLRP1 is involved in acquired drug resistance to TMZ in melanoma.

Interestingly, increased NLRP1 expression and IL-1β secretion in both TMZ-treated parental and acquired resistant cells were linked to the Notch signaling pathway. Notch1 activation was shown to promote tumor growth and drive acquired resistance to mitogen-activated protein kinase (MAPK) inhibitors in melanoma [38], as well as TMZ resistance in melanoma [39] and glioma [31,40]. Notch signaling regulates the self-renewal and survival of cancer stem cells, which are intimately related to tumor initiation and chemoresistance [31]. Because Notch has many downstream targets that are involved in apoptosis, cell cycle, and cell survival, this protein could be responsible for acquired resistance to various drugs or cross-resistance to therapy options with different mechanisms of action [30,31,38,39,40,41,42]. Although the exact mechanisms underlying the relationship between Notch signaling and NLRP1 are unknown, the crosstalk between Notch signaling and other pathways, including MAPK, NF-κB, and IL-1β signaling [30], was observed and may indirectly explain the interaction between Notch and NLRP1. Besides, NLRP1 participates in many cellular processes through pyrin domain (PYD)-PYD and CARD-CARD protein interactions [7,43]; therefore, this inflammasome sensor protein could be involved in non-inflammasome pathways, connecting NLRP1 to cross-resistant mechanisms to various drug treatments, as well as stemness phenotype.

**Figure 8:**
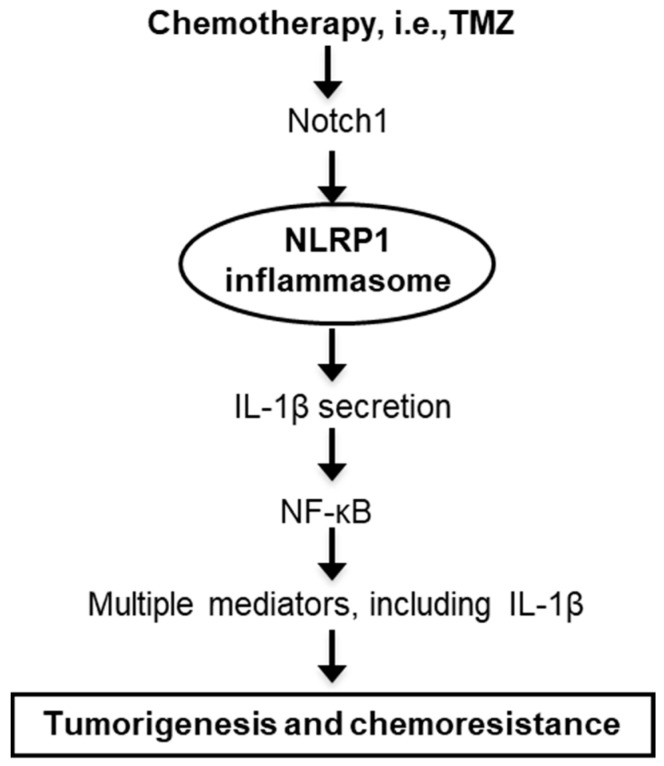
Flow-chart model proposal to explain the NLRP1 involvement in melanoma chemoresistance. NLRP1 is a component of the activated and NF-κB-associated signaling pathways responsible for resistance following chemotherapeutic assaults.

Strategies to overcome drug resistance and enhance drug sensitivity include combination therapies using agents that target known resistance mechanisms. TMZ has been combined with inhibitors that target not only MGMT [15], NF-κB [13], and Notch signaling [39] but also AKT [44], mTOR signaling [45], autophagy [46,47], and glutaminase-mediated glutamine metabolism [48], all of which significantly enhanced TMZ cytotoxicity in melanoma cells. Additional work in glioma demonstrated improved therapeutic results from the concurrent use of TMZ and c-Jun N-terminal kinase inhibitors [49]. Cytokines can also be used to overcome drug resistance. A tumor suppressor cytokine IL-24 is downregulated during melanoma progression, and supplementing IL-24 has shown to reverse melanoma resistance to TMZ by downregulating p53 and MGMT activity [25]. Our current work on NLRP1 would, therefore, add another therapeutic target to the list. Notch pathway inhibitors, including γ-secretase inhibitor, Notch 1 siRNA, and monoclonal antibodies against Notch receptors and ligands, are currently in clinical trials for numerous cancers [41]. Because NLRP1 expression and IL-1β secretion are downstream mediators of TMZ-induced Notch activation (Figure 8), inhibiting NLRP1 and/or IL-1β could be used to regulate cancer aggressiveness and drug resistance, as we have shown in the current study.

Additionally, our current work on the involvement of NLRP1 inflammasome in acquired drug resistance could help tailor melanoma treatment with non-chemotherapy options, such as targeted therapies. As a pleiotropic pro-inflammatory cytokine, IL-1β is involved in various malignant processes, including upregulating key oncogenic players and maintaining tumor immunosuppressive microenvironment [50,51]. Although we do not know yet the mechanism by which TMZ enhances NLRP1 expression and inflammsome activation, TMZ treatment likely activates an off-target unfolded protein response, resulting in increased levels of the activating transcription factor 4 (ATF4) [52,53], which regulates NLRP1 expression [54] and subsequent IL-1β secretion. Indeed, we demonstrated that NLRP1 plays a role in vemurafenib resistance in melanoma through transcriptional regulation by ATF4, in which activation is mediated by the MAPK/ extracellular signal-regulated kinase pathway [55]. These findings may explain the observations of increased IL-1β levels in reactivated, vemurafenib-resistant melanoma cells [55].

Furthermore, the current work could extend our understanding of drug resistance in other non-chemotherapy drugs. The recent introduction of immunotherapy into melanoma treatment has rapidly changed the clinical strategy for melanoma; however, only a limited number of patients benefit. Accordingly, a combination strategy has been tried to improve the outcome, such as using chemotherapy and targeted therapies to increase tumor-specific antigen release and presentation [56]. So far, the combination of checkpoint blockade and TMZ therapy has yielded a promising anti-tumor efficacy in experimental models and clinical trials [57,58]. However, unlike vemurafenib, which reduces tumor-associated inflammation [50], TMZ not only enhances inflammatory response as observed in our current study but also promotes immune escape by upregulating PD-L1/L2 expression and reducing lymphocyte infiltration [57,59], thus potentially favoring a therapeutic response to immunotherapy [60].

Inflammasome sensor proteins and IL-1 blocking therapies are expanding in humans [61,62,63,64,65], and, as a result, we will be able to more adequately control cancers that have activated NLRP or secrete IL-1β than those cancers that do not. Given the safety profile and clinical availability of IL-1 blocking agents, such as IL-1Ra, anti-IL-1β monoclonal antibody, IL-1 Trap, oral caspase-1 inhibitor, and NLRP inhibitors [61,62,63,66], our promising findings could easily be translated into patient therapies.

## 4. Materials and Methods

### 4.1. Chemicals

TMZ was purchased from Sigma (T2577; St. Louis, MO, USA), reconstituted at a stock concentration of 100 mM in dimethyl sulfoxide (DMSO), and stored at −20 °C in aliquots. The γ-secretase inhibitor, DAPT, was purchased from Abcam (ab120633; Cambridge, MA, USA), reconstituted at a stock concentration of 50 mM in DMSO, and stored at −20 °C until use. Ready-to-use actinomycin D was purchased from BioVision (K121; Milpitas, CA, USA) and stored at −20 °C. Kineret (Anakinra, recombinant human IL-1 receptor antagonist, IL-1Ra) was purchased from Swedish Orphan Biovitrum AB (Stockholm, Sweden) and kept at 4 °C. Features and origin of other chemicals and reagents are indicated elsewhere.

### 4.2. Cell Culture

Human metastatic melanoma cell lines were obtained from the American Type Culture Collection (Manassas, VA, USA) and grown at 37 °C in RPMI 1640 supplemented with 10% fetal bovine serum (Gemini Bioproducts, West Sacramento, CA, USA), 100 I.U/mL penicillin, and 100 μg/mL streptomycin (Mediatech, Manassas, VA, USA) in a 5% CO_2_ incubator. Cells were regularly monitored for mycoplasma contamination using PCR [67]. All cell lines have been authenticated using short tandem repeat (STR) fingerprinting by the Barbara Davis Center BioResource Core at the University of Colorado Anschutz Medical Campus.

### 4.3. Cell Growth Inhibition

To evaluate the sensitivity to TMZ, cells were seeded into a 96-well plate at a density of 1–2 × 10^3^ cells per well and allowed to adhere for at least 2 h before being exposed to increasing concentrations of TMZ in triplicate for 3–4 days. Cell growth inhibition was determined using the CellTiter 96 Aqueous One Solution Cell Proliferation Assay kit (G3580; Promega, Madison, WI, USA), and, in the experiments of developing TMZ-resistant cell lines, the IC_20_ or IC_50_ value (TMZ concentration inhibiting 20 or 50% cell growth) was measured.

### 4.4. Generation of Acquired Resistance to TMZ

Previous studies performed with 6 melanoma cell lines tested TMZ IC_50_ concentrations of 250–800 µM [68]. Since in clinical settings, the peak plasma levels of TMZ after oral administration of a single dose reach approximately 80 µM [22], an IC_20_ dose of TMZ that falls within its pharmacological range was used to generate resistant cell lines. Cells were plated into a 100 mm dish at a density of 1.2–2 × 10^5^ cells per dish and treated with the IC_20_ dose of TMZ. The medium was refreshed with TMZ every 2–3 days, and cells were passaged at confluency. After 3–4 passages, IC_20_ values were re-evaluated, and cells were treated with the escalated IC_20_ doses. 1205Lu and HS294T resistant cell lines were thus generated after continuously treated with increasing doses of TMZ for over 2 months. TMZ-acquired resistance was defined as a four-fold increase of IC_50_ over that of parental cells [69]. Figure S7 shows that the resistance phenotype of 1205Lu persists after a freeze-thaw cycle and then TMZ withdrawal for one month. Resistant cells were regularly maintained in culture medium with 100 µM TMZ.

### 4.5. NLRP1 and NLRP3 Knockdown

Cells seeded in appropriate culture plates were transfected overnight at 60% confluency with 40 nM NLRP1 FlexiTube GeneSoluion siRNA and/or NLRP3 FlexiTube siRNA (both used a mixture of two preselected siRNAs) or negative control (Qiagen, Valencia, CA, USA) using Lipofectamine 2000 (#11668-019; Invitrogen, Carlsbad, CA, USA) in Opti-MEM 1 reduced serum medium (#31985-070; Gibco, Grand Island, NY, USA). Cells with transient knockdown of NLRP1 and/or NLRP3 were used in the experiments as indicated.

### 4.6. Quantitative RT-PCR

Total RNA was isolated using RNeasy Plus Mini kit (#74136; Qiagen, Hilden, Germany) and used for reverse transcription reactions with MMLV reverse transcriptase (M1701; Promega) or iScript cDNA synthesis kit (Bio-Rad Laboratories, Hercules, CA, USA). Quantitative PCR was performed using the Power SYBR Green PCR Master Mix (A25778; Applied Biosystems, Foster City, CA, USA) on the MX3000P PCR system (Applied Biosystems) or AriaMx real-time PCR system (Agilent Technologies, Santa Clara, CA, USA). The primers used are listed in Table S1.

### 4.7. Western Blot

Cells were lysed in RIPA buffer containing 1% protease inhibitor cocktail (Sigma). To assess the localization of NF-κB p65, cytoplasmic and nuclear fractions of cells were isolated using NE-PER Nuclear and Cytoplasmic Extraction Reagents (#78833; Thermo Scientific, Rockford, IL, USA). Equal amounts of cellular proteins were separated on Novex 4–20% Tris-Glycine gels (Life Technologies, Carlsbad, CA, USA) or Mini-PROTEAN TGX precast gels (Bio-Rad Laboratories), followed by electrotransfer onto a PVDF membrane (Pall Corporation, Pensacola, FL, USA). After blocking with 5% nonfat milk, the immunoblot was incubated with a primary antibody, washed, and then incubated with the appropriate species-specific horseradish peroxidase-conjugated secondary antibody (Sigma). The primary antibodies include rabbit anti-MGMT (A0693, 1:1000; Neo Scientific, Woburn, MA, USA), rabbit anti-caspase-3 (detecting full length and cleavage fragment) (#9662, 1:1,000; Cell Signaling Technology, Danvers, MA, USA), rabbit anti-cleaved caspase-3 (#9664, 1:1000; Cell Signaling Technology), rabbit anti-PARP (#9542, 1:1250; Cell Signaling Technology), mouse anti-NLRP1 (ALX-804-803, 1:1,000; Enzo Life Sciences, Farmingdale, NY, USA), rabbit anti-NLRP3 (#13158, 1:1,250; Cell Signaling Technology), rabbit anti-caspase-1 (detecting endogenous levels of pro-caspase-1 and p20 caspase-1) (#2225, 1:1000; Cell Signaling Technology), rabbit anti-cleaved caspase-1 (#4199, 1:1000; Cell Signaling Technology), mouse anti-NF-κB p65 (sc-8008, 1:500; Santa Cruz Biotechnology, Santa Cruz, CA, USA), goat anti-Lamin B (sc-7217, 1:500; Santa Cruz Biotechnology), rabbit anti-cyclophilin A (#2175, 1:1250; Cell Signaling Technology), goat anti-Notch1 (sc-6014, 1:500; Santa Cruz Biotechnology), rabbit anti-GAPDH (#PA1-987, 1:1000; Invitrogen), mouse anti-β-actin (A2228, 1:2000; Sigma), and rabbit anti-β-actin (sc-1616-R, 1:1,000; Santa Cruz Biotechnology). Signals were visualized by SuperSignal West Femto Maximum Sensitivity Substrate (#34096; Thermo Scientific) and analyzed using the Odyssey imaging system (LI-COR, Lincoln, NE, USA).

### 4.8. Enzyme-Linked Immunosorbent Assay (ELISA)

The human IL-1β ELISA kit was obtained from R&D Systems (#DY201; Minneapolis, MN, USA) and used according to manufacturer’s instructions. Culture supernatants from cells incubated in Opti-MEM 1 for 18 h were collected and assayed for secreted IL-1β, while intracellular pro-IL-1β was assayed by lysing cells with 0.5% Triton X-100 in phosphate-buffered saline followed by two freeze-thaw cycles [9].

### 4.9. Caspase-1 Activity Assay

Caspase-1 activity was measured using a FAM-FLICA (fluorochrome-labeled inhibitors of caspases) caspase assay kit (#97; ImmunoChemistry Technologies, Bloomington, MN, USA) and a Synergy 2 multi-mode microplate reader (BioTek, Winooski, VT, USA).

### 4.10. Lactate Dehydrogenase (LDH) Assay

The release of LDH into the culture medium as a marker of cell death was determined using a Pierce LDH cytotoxicity assay kit (#88954; Pierce Biotechnology, Rockford, IL, USA).

### 4.11. NF-κB Activity Assay

Cells seeded in a 24-well plate at 5 × 10^4^ cells/well overnight were transfected with a control vector (pMetLuc2) or an NF-κB reporter vector (pNF-κB-MetLuc2) (#631743; Clontech Laboratories, San Francisco, CA, USA) using Lipofectamine 2000 for 4.5 h, and then cell culture medium was added. Supernatants were harvested 18 h after the transfection and analyzed for NF-κB activity using the Ready-to-Glow Secreted Luciferase Vector kit (#631727; Clontech Laboratories) according to manufacturer’s instructions.

### 4.12. Tumor Formation In Vivo

1 × 10^6^ 1205Lu cells were suspended in Matrigel Matrix (BD Biosciences, San Jose, CA, USA) diluted 1:1 with phosphate-buffered saline and injected subcutaneously into both left and right flanks of eight-week-old female athymic nu/nu mice from Jackson Laboratories (Bar Harbor, ME, USA). Tumor growth was monitored at least twice a week with an electronic digital caliper, and tumor volume was calculated according to the formula: tumor volume (mm^3^) = longest diameter × shortest diameter^2^/2. When tumor volume reached 100 mm^3^, mice were randomly assigned into different groups and treated intraperitoneally with 15 mg/kg TMZ or 10% DMSO as vehicle control daily for consecutive 5 days. When indicated, mice were subcutaneously treated daily with 30 mg/kg IL-1Ra or saline, starting on the first day of the TMZ treatment, until the end of the experiment. Mice were then sacrificed, and tumors were harvested. Tumor tissues were analyzed histologically or subjected to single-cell isolation for NLRP1 expression analysis, as previously described [70]. All experimental manipulations were approved by the Institutional Animal Care and Use Committee of the University of Colorado Anschutz Medical Campus, Aurora, CO, on 9th October 2017 under the protocol number 00282.

### 4.13. Immunohistochemistry

Tumor tissues were harvested and fixed in 10% neutral buffered formalin. After paraffin embedding, tumor specimens were cut into 5-μm sections and stained with hematoxylin and eosin. NLRP1, IL-1β, Notch1, Ki-67, and caspase-3 staining were performed following the standard protocols using mouse anti-NLRP1 (ALX-804-803, 1:200; Enzo Life Sciences), Goat anti-IL-1β (#AF-201-NA, 1:200; R&D Systems), goat anti-Notch1 (sc-6014, 1:200; Santa Cruz Biotechnology), rabbit anti-Ki-67 (ab15580, 1:500; Abcam, Cambridge, MA, USA), and rabbit anti-caspase-3 (#9662, 1:1,000; Cell Signaling Technology), respectively, diluted in Dako antibody diluent (Dako North America, Carpinteria, CA, USA), followed by N-Histofine Simple Stain MAX PO (Multi) (#414152F; Nichirei Biosciences, Tokyo, Japan). Sections were counterstained with hematoxylin and reviewed by two observers.

### 4.14. Statistical analysis

GraphPad Prism 7 was used for statistical analysis. Differences between treatment groups were analyzed by one-way ANOVA with Bonferroni’s or Dunnett’s post-tests. A value *p* < 0.05 was considered statistically significant.

## 5. Conclusions

In this paper, we demonstrated a novel mechanism of drug resistance where activated NLRP1 inflammasomes contribute to TMZ-induced acquired resistance in metastatic melanoma. This is the first report to demonstrate the involvement of NLRP in the development of acquired drug resistance and support the application of novel resistance mechanism-targeted inhibitors as a strategy to improve the efficacy of current therapeutic agents.

## Supplementary Materials

The following are available online at https://www.mdpi.com/2072-6694/12/9/2518/s1. Figure S1: Original blots of Figure 1B, Figure S2: Original blots of Figure 1D, Figure S3: Original blots of Figure 1E, Figure S4: Original blots of Figure 1F, Figure S5: (A) Original blots of Figure 2B. (B) Original blots of Figure 2B (cont’d), Figure S6: Original blots of Figure 2F, Figure S7. NF-κB activity in A375 and SK-ME-28 cells treated with 100 µM TMZ for 48 h, Figure S8. IL-1β production in A375 cells treated with 100 µM TMZ for 48 h, Figure S9: Original blots of Figure 4A, Figure S10: 1205Lu cells retain the resistant phenotype after being subjected to a freeze-thaw cycle and TMZ withdrawal for one month, Figure S11: Original blots of Figure 4C, Figure S12: Original blots of Figure 4D, Figure S13: Original blots of Figure 4E, Figure S14: Knocking down NLRP1 has no effect on the proliferation of resistant cells without TMZ treatment, Figure S15: IL-1Ra does not affect the proliferation of resistant 1205Lu cells in the absence of TMZ, Figure S16. IL-1Ra does not alter the sensitivity of primarily resistant SK-MEL-28 cells to TMZ treatment, Figure S17: Original blots of Figure 5A, Figure S18: Original blots of Figure 5B, Figure S19: NLRP1 inflammasomes are not required for Notch1 upregulation by TMZ treatment or in TMZ-resistant cells. Table S1. Primers used for quantitative RT-PCR.
