# Supplementary material for: Inflammasome Sensor NLRP1 Confers Acquired Drug Resistance to Temozolomide in Human Melanoma
Source: Cancers (Basel). 2020 Sep 4;12(9):2518. doi: 10.3390/cancers12092518 (PMC7563249; doi:10.3390/cancers12092518)
Supplement: Supplementary file 1 [file cancers-12-02518-s001.pdf]

Supplementary Materials

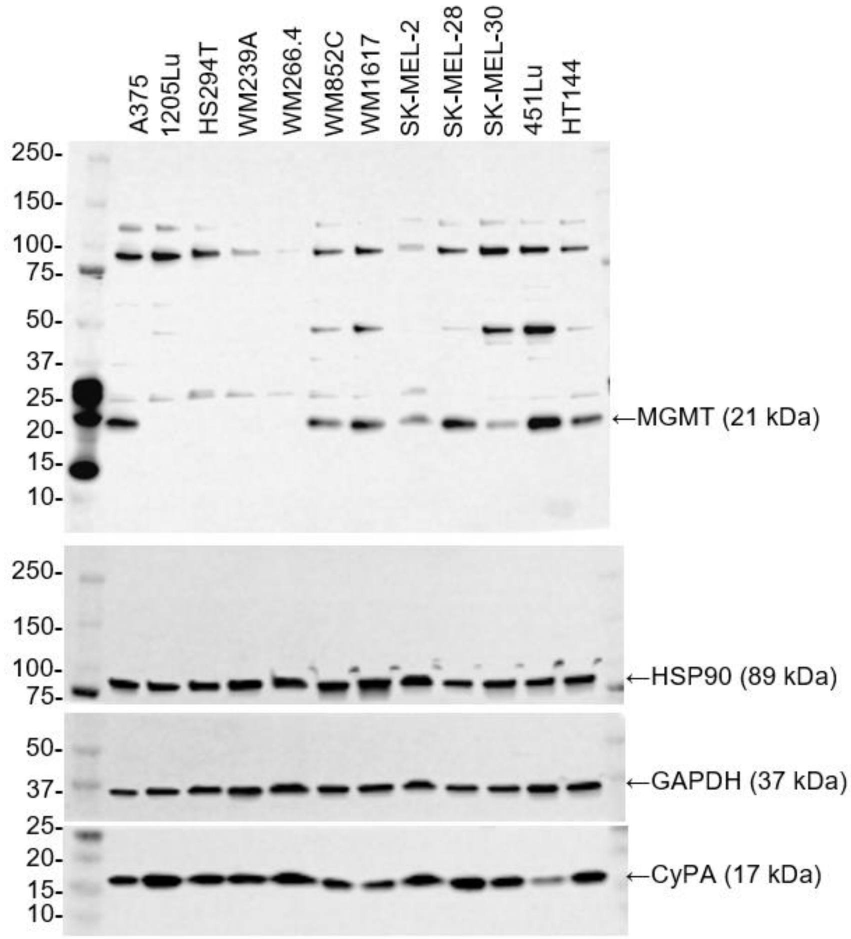

Figure S1. Original blots of Figure 1B.

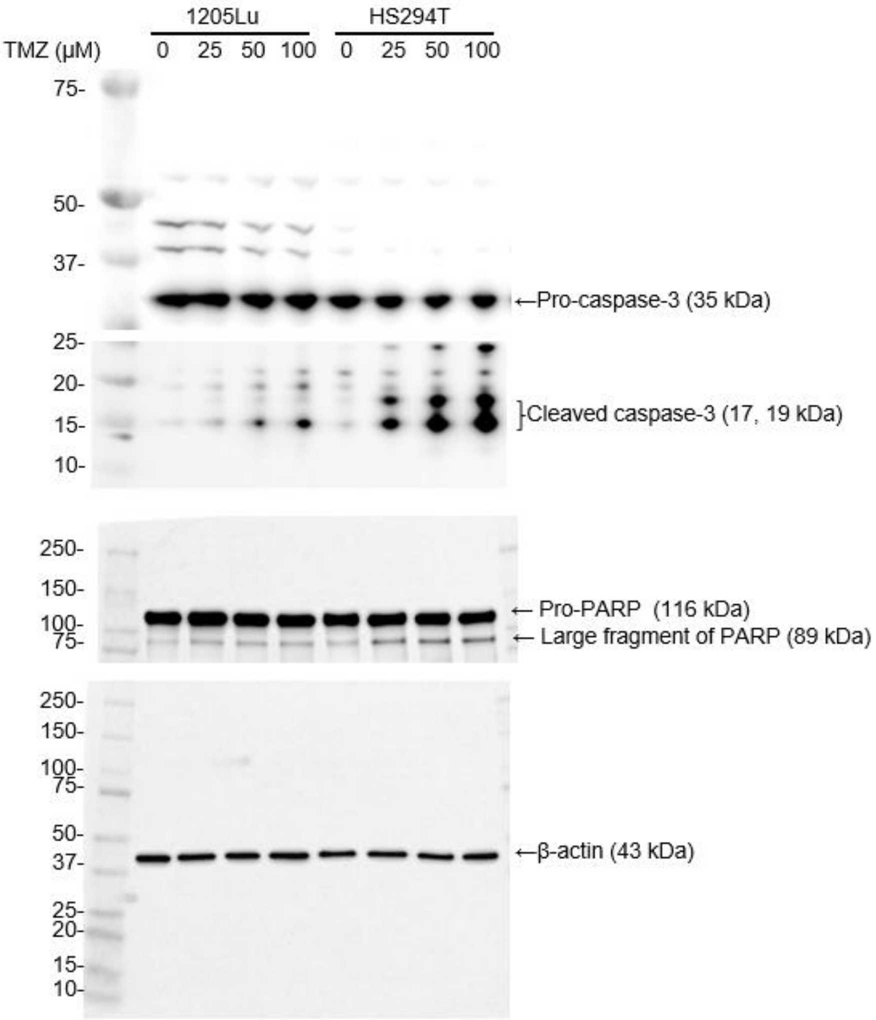

Figure S2. Original blots of Figure 1D.

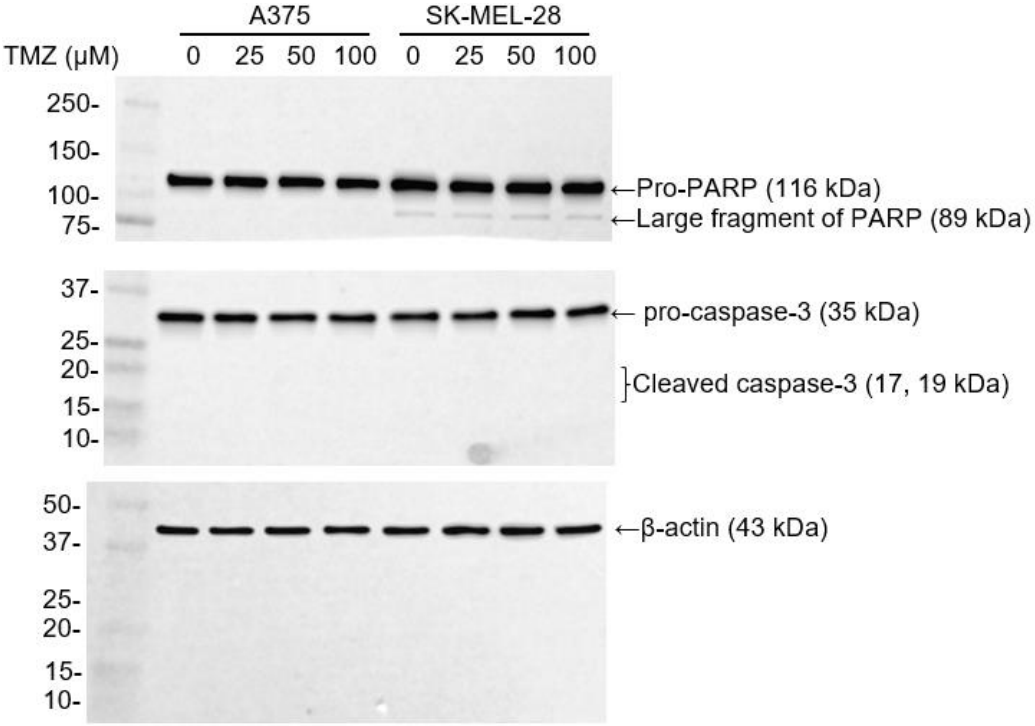

Figure S3. Original blots of Figure 1E.

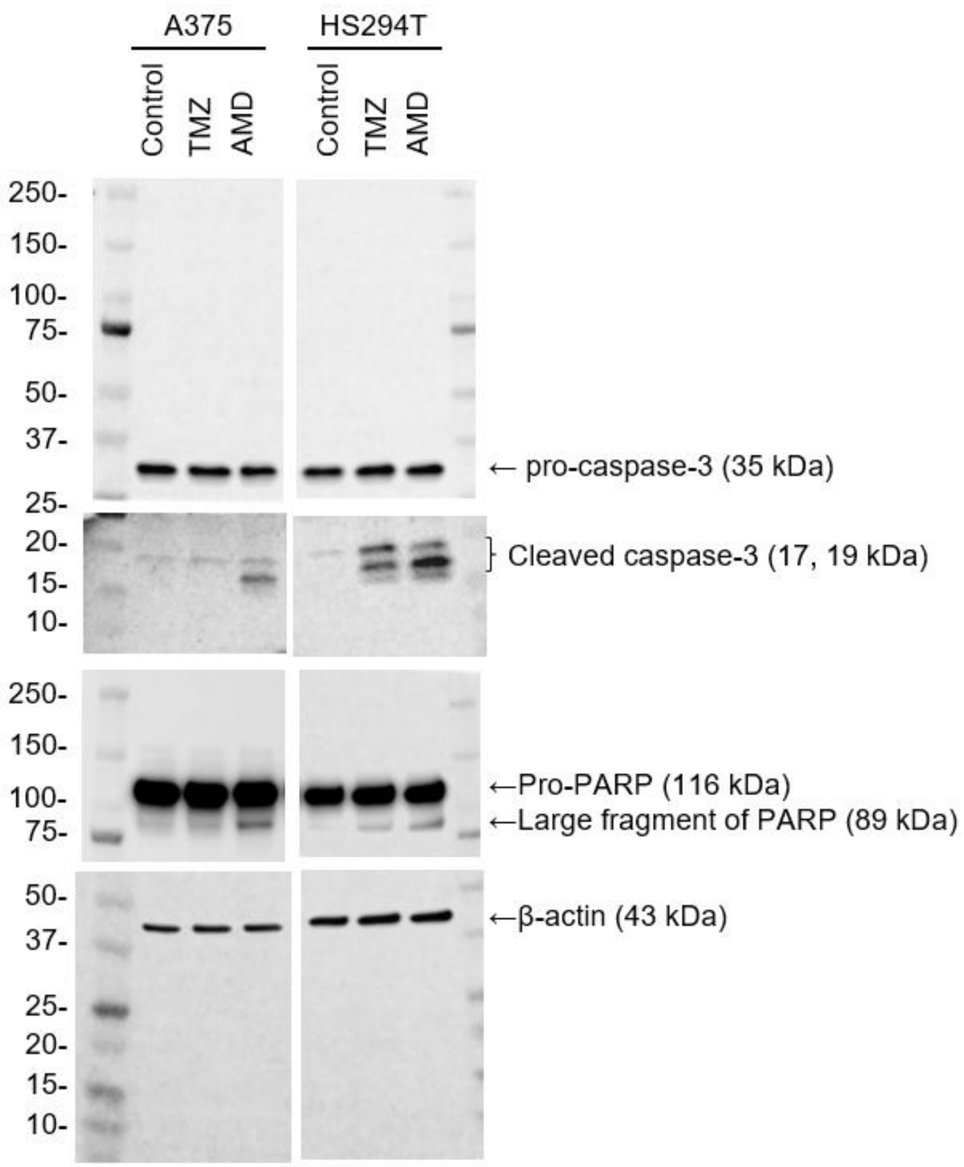

Figure S4. Original blots of Figure 1F.

A

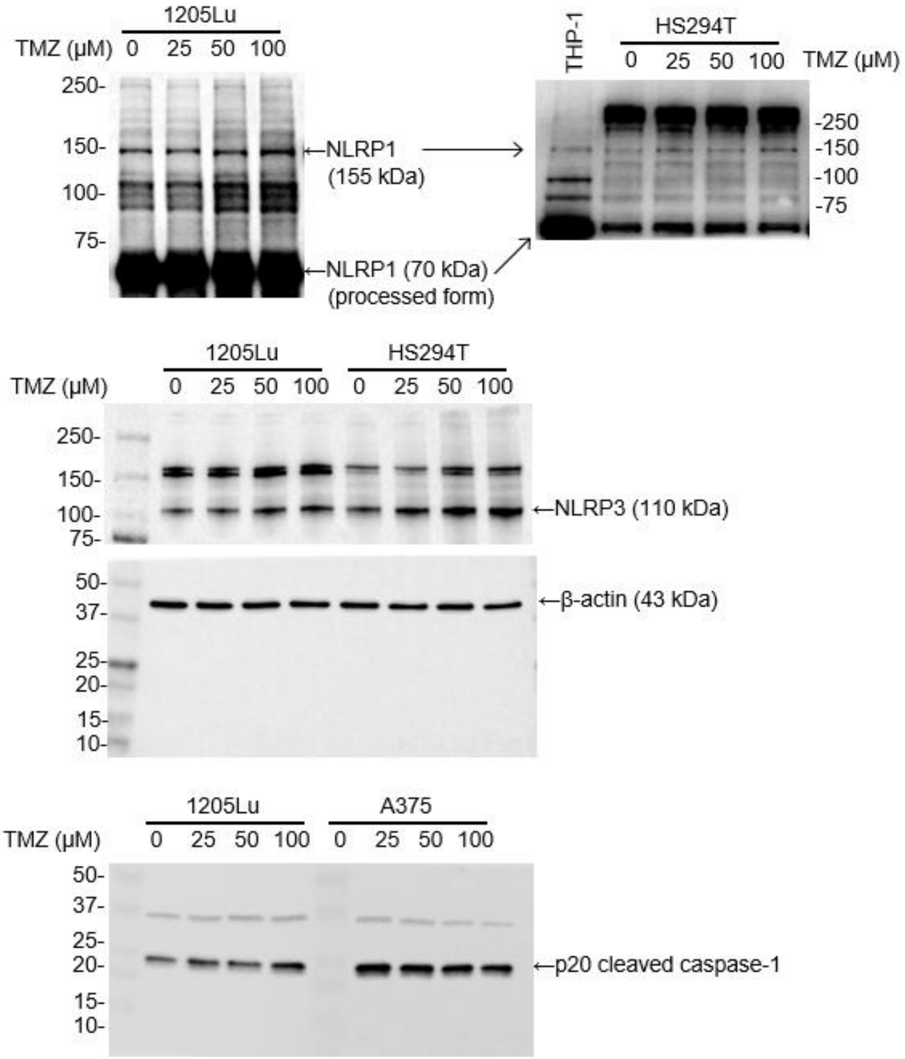
1205Lu
TMZ (μM) 0 25 50 100
250-
150-
100-
75-
←NLRP1 (155 kDa)
←NLRP1 (70 kDa) (processed form)
THP-1
HS294T
0 25 50 100 TMZ (μM)
-250
-150
-100
-75
1205Lu
HS294T
TMZ (μM) 0 25 50 100 0 25 50 100
250-
150-
100-
75-
←NLRP3 (110 kDa)
50-
37-
25-
20-
15-
10-
←β-actin (43 kDa)
1205Lu
A375
TMZ (μM) 0 25 50 100 0 25 50 100
50-
37-
25-
20-
15-
10-
←p20 cleaved caspase-1

B

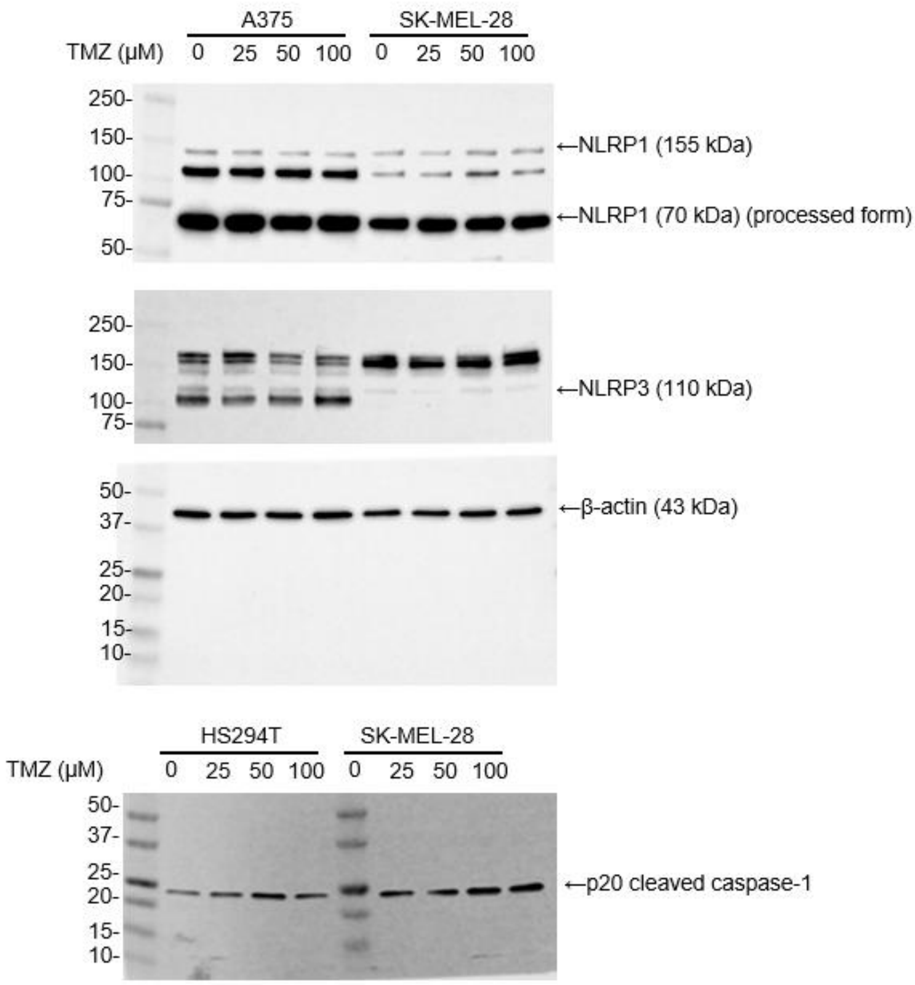

Figure S5. (**A**) Original blots of Figure 2B. (**B**) Original blots of Figure 2B (cont'd).

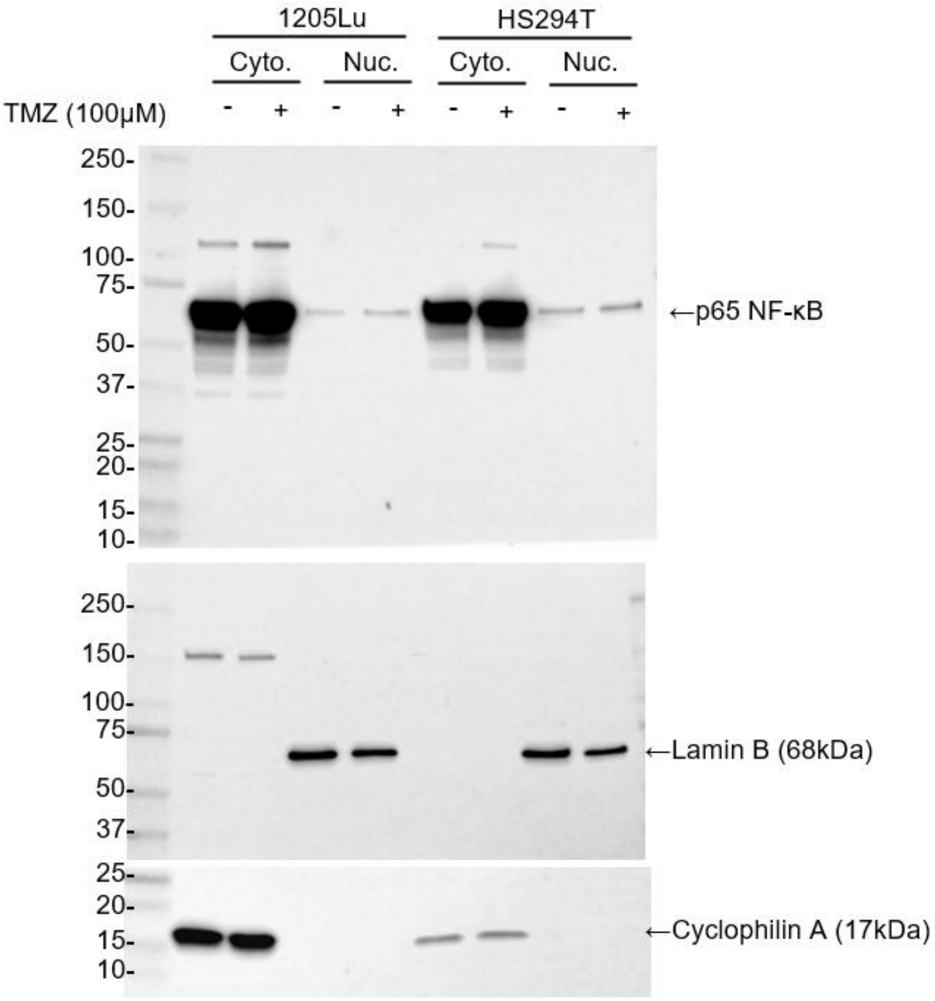

Figure S6. Original blots of Figure 2F.

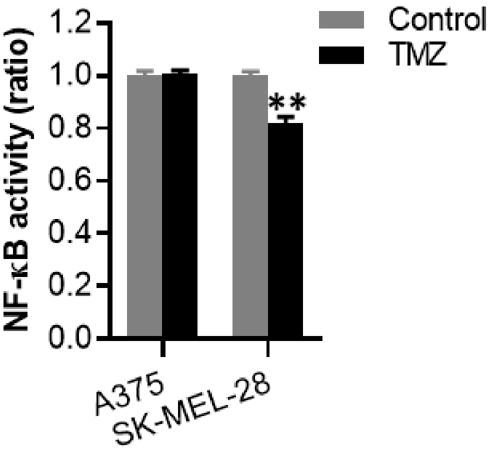

Figure S7. NF-κB activity in A375 and SK-ME-28 cells treated with 100 μM TMZ for 48 h. ** $p < 0.01$.

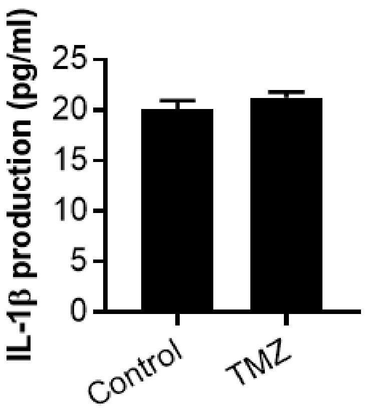

Figure S8. IL-1β production in A375 cells treated with 100 μM TMZ for 48 h.

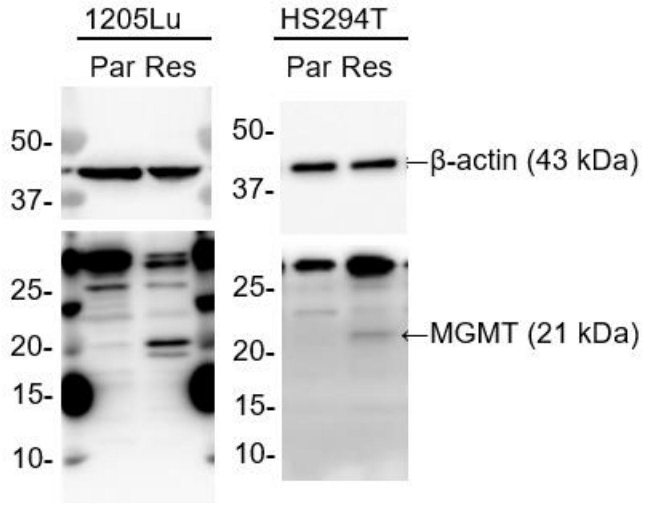

Figure S9. Original blots of Figure 4A.

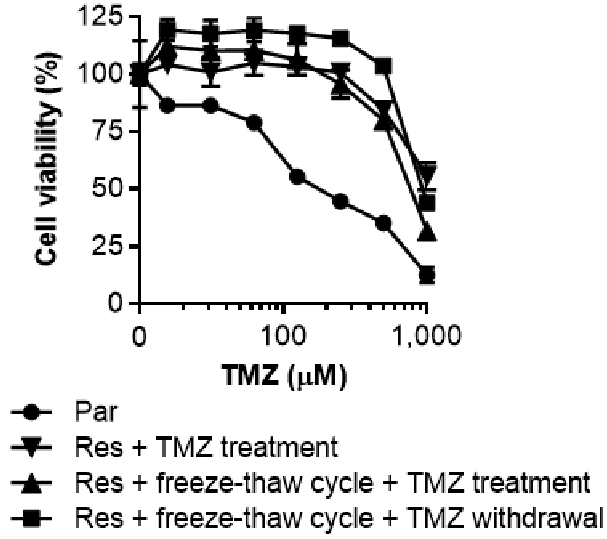

Figure S10. 1205Lu cells retain the resistant phenotype after being subjected to a freeze-thaw cycle and TMZ withdrawal for one month. 1205Lu parental cells (Par), resistant cells treated with TMZ (Res + TMZ treatment), resistant cells with a freeze-thaw cycle and TMZ treatment (Res + freeze-thaw cycle + TMZ treatment), and resistant cells with a freeze-thaw cycle and then TMZ withdrawal for one month (Res + freeze-thaw cycle + TMZ withdrawal) were treated with the indicated TMZ doses for three days. Then, cell viability was determined by MTS assay. Mean ± SEM (*n* = 2-4).

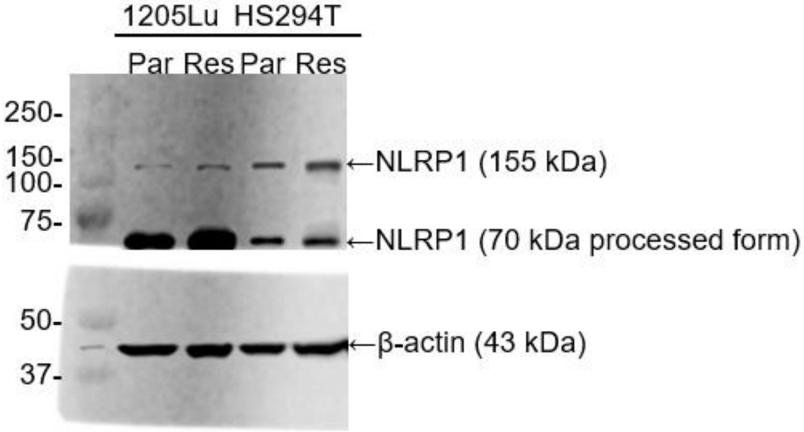

Figure S11. Original blots of Figure 4C.

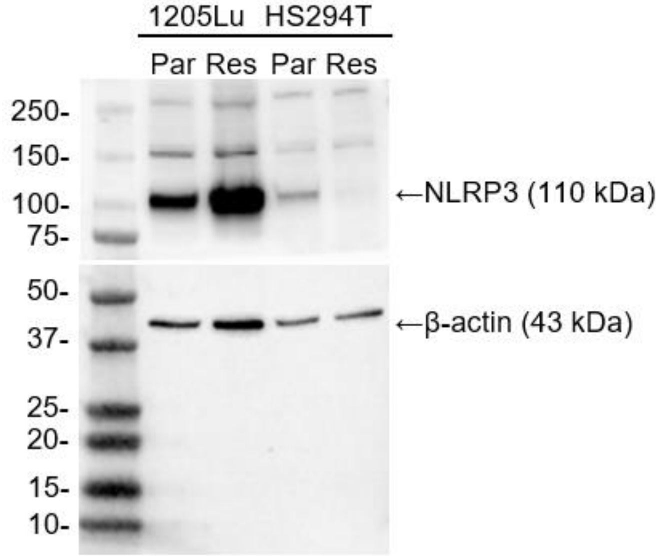

Figure S12. Original blots of Figure 4D.

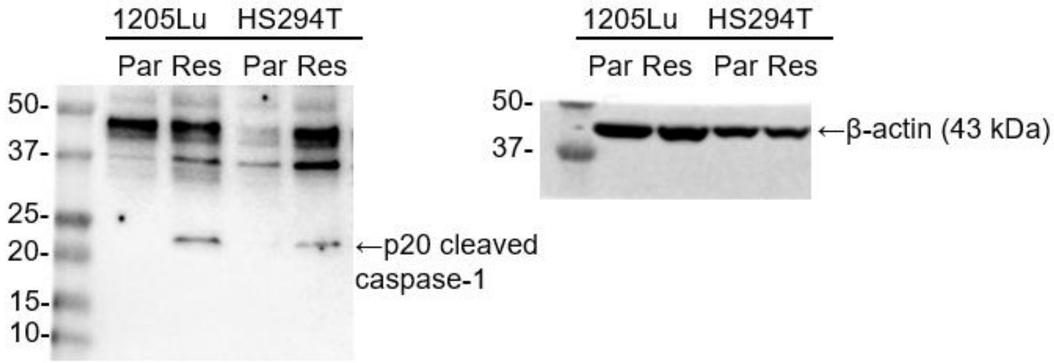

Figure S13. Original blots of Figure 4E.

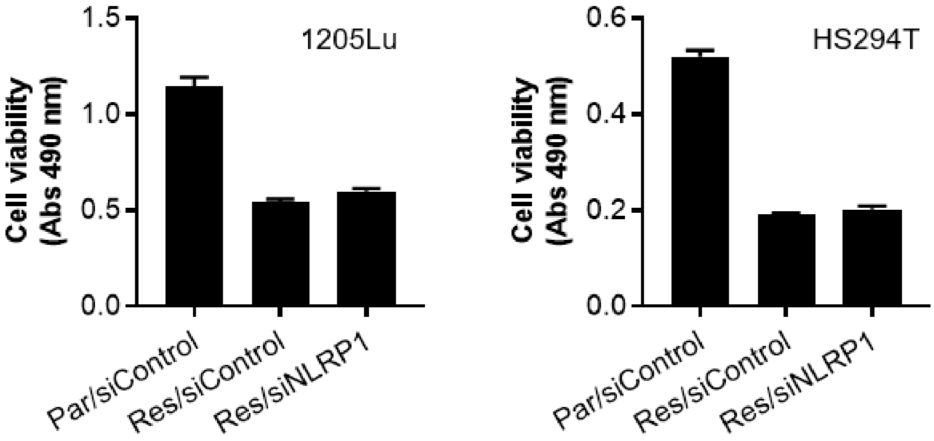

Figure S14. Knocking down NLRP1 has no effect on the proliferation of resistant cells without TMZ treatment. Cells were transfected with NLRP1 siRNA mixture (40 nM) overnight, then seeded in 96-well plates at low density with TMZ for 3-4 days. Cell viability was determined by MTS assay. See **Figure 4G** for NLRP1 knockdown efficiency. Mean ± SEM (n = 3).

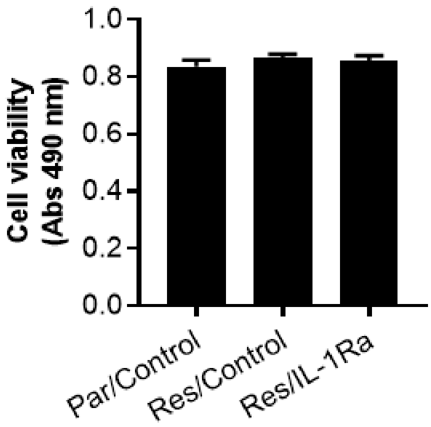

Figure S15. IL-1Ra does not affect the proliferation of resistant 1205Lu cells in the absence of TMZ. Cells were treated with IL-1Ra (10 μg/mL) daily for 3 days, then cell viability was determined by MTS assay. Mean ± SEM (*n* = 4).

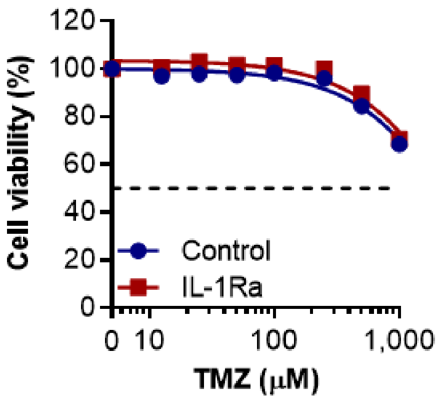

Figure S16. IL-1Ra does not alter the sensitivity of primarily resistant SK-MEL-28 cells to TMZ treatment. Cells were treated with IL-1Ra (10 μg/mL) and TMZ daily for 3 days, then cell viability was determined by MTS assay. Mean ± SEM (*n* = 3).

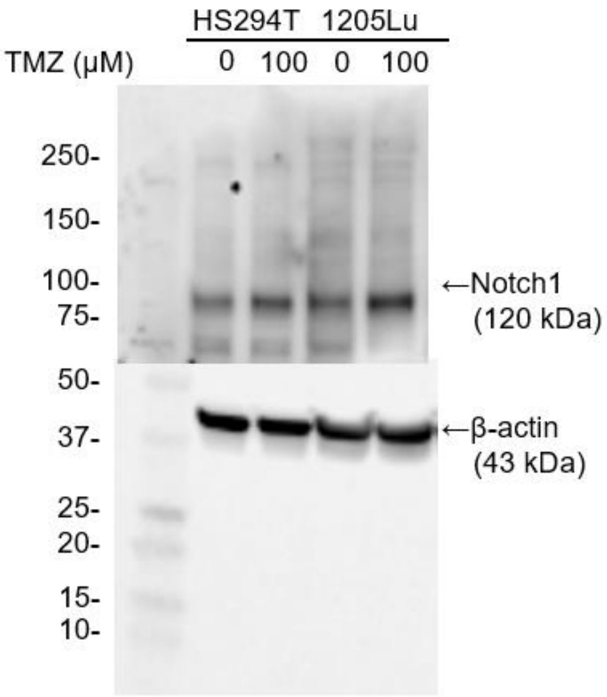

Figure S17. Original blots of Figure 5A.

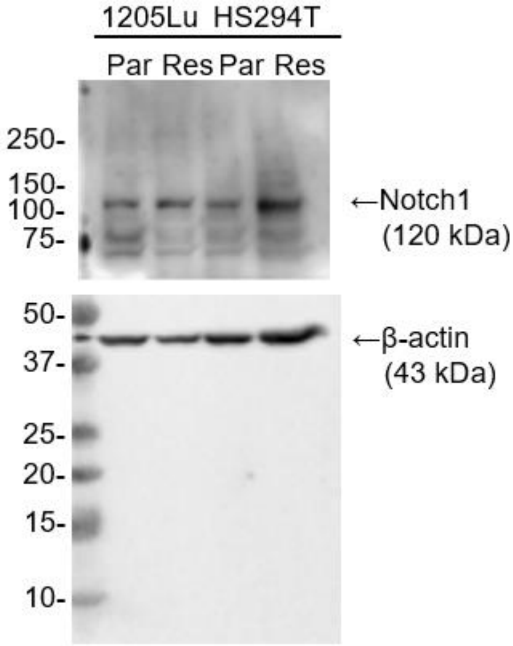

Figure S18. Original blots of Figure 5B.

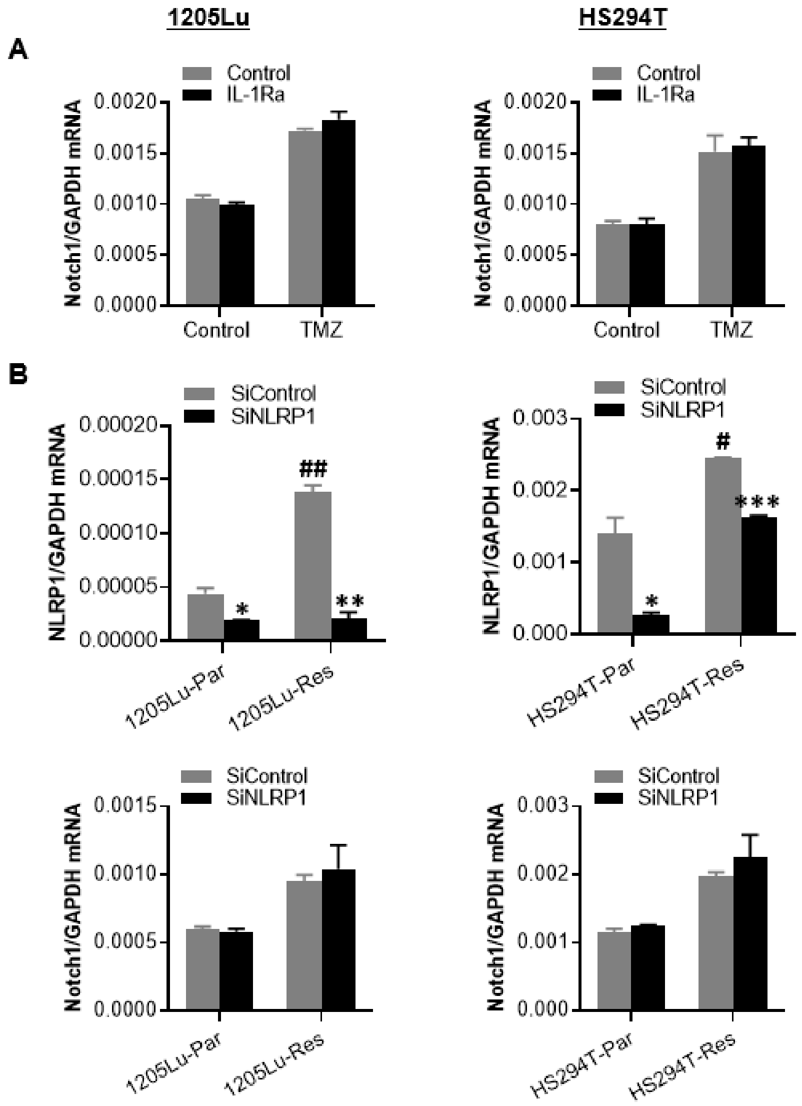

Figure S19. NLRP1 inflammasomes are not required for Notch1 upregulation by TMZ treatment or in TMZ-resistant cells. (**A**) Parental 1205Lu and HS294T cells were treated with 10 µg/mL IL-1Ra and/or 100 µM TMZ for 24 h, then Notch1 mRNA expression was analyzed by qRT-PCR. (**B**) Parental (Par) and resistant (Res) cells were transfected with 40 nM siRNA control or NLRP1 siRNA mixture for 48 h, then Notch1 gene expression was assessed. Top panels show the gene silencing effects of NLRP1 in parental and resistant cells. Mean ± SEM (n = 3). * $p < 0.05$, ** $p < 0.01$, and *** $p < 0.001$ vs. the corresponding siControl; $^{\#}p < 0.05$ and $^{\#\#}p < 0.01$ vs. parental cells with siControl.

Table S1. mRNA primers for quantitative RT-PCR.

Gene	Forward 5′-3′	Reverse 53′
MGMT	CCCGCGCCCCGGATA	CAAGTACCAAGTCGCAAACGG
NLRP1	GCTGGACCAGACAACTCTGA	GGTTTCCGTCTGCTGAAGAT
NLRP3	GATCTTCGCTGCGATCAACAG	CGTGCATTATCTGAACCCCAC
Notch1	GACAGCCTCAACGGGTACAA	CACACGTAGCCACTGGTCAT
GAPDH	CAGGGCTGCTTTTAACTCTGG	TGGGTGGAATCATATTGGAACA
